# Synthesis of Highly Branched Polyolefins Using Phenyl Substituted α-Diimine Ni(II) Catalysts

**DOI:** 10.3390/polym8040160

**Published:** 2016-04-22

**Authors:** Fuzhou Wang, Ryo Tanaka, Zhengguo Cai, Yuushou Nakayama, Takeshi Shiono

**Affiliations:** 1 Graduate School of Engineering, Hiroshima University, Kagamiyama 1-4-1, Higashi-Hiroshima 739-8527, Japan; wangfuzhou1718@126.com (F.W.); rytanaka@hiroshima-u.ac.jp (R.T.); yuushou@hiroshima-u.ac.jp (Y.N.); 2 State Key Lab of Chemical Fibers & Polymer Materials, College of Material Science & Engineering, Donghua University, Shanghai 201620, China; caizg@dhu.edu.cn

**Keywords:** α-diimine Ni(II) complex, chain-walking polymerization, branched polyolefins, living polymerization, 1-alkene

## Abstract

A series of α-diimine Ni(II) complexes containing bulky phenyl groups, [ArN = C(Naphth)C = NAr]NiBr_2_ (Naphth: 1,8-naphthdiyl, Ar = 2,6-Me_2_-4-PhC_6_H_2_ (**C1**); Ar = 2,4-Me_2_-6-PhC_6_H_2_ (**C2**); Ar = 2-Me-4,6-Ph_2_C_6_H_2_ (**C3**); Ar = 4-Me-2,6-Ph_2_C_6_H_2_ (**C4**); Ar = 4-Me-2-PhC_6_H_3_ (**C5**); Ar = 2,4,6-Ph_3_C_6_H_2_ (**C6**)), were synthesized and characterized. Upon activation with either diethylaluminum chloride (Et_2_AlCl) or modified methylaluminoxane (MMAO), all Ni(II) complexes showed high activities in ethylene polymerization and produced highly branched amorphous polyethylene (up to 145 branches/1000 carbons). Interestingly, the *sec-*butyl branches were observed in polyethylene depending on polymerization temperature. Polymerization of 1-alkene (1-hexene, 1-octene, 1-decene and 1-hexadecene) with **C1-**MMAO at room temperature resulted in branched polyolefins with narrow *M*_w_/*M*_n_ values (*ca.* 1.2), which suggested a living polymerization. The polymerization results indicated the possibility of precise microstructure control, depending on the polymerization temperature and types of monomers.

## 1. Introduction

Polymerization of ethylene and 1-alkenes plays pivotal roles in today’s polymer industry, because polyolefins are tremendously important in everyday life [1]. Since Brookhart and co-workers discovered Ni(II) and Pd(II) aryl-substituted α-diimine complexes for alkene polymerization [2,3,4,5,6], late transition metal catalysts have attracted interesting attention due to their high functionality. Living/controlled alkene polymerization is important to synthesize polymers with predictable molecular weights and narrow molecular weight distributions, end-functionalized polymers and well-defined block copolymers [7,8,9,10,11,12,13].

**Scheme 1:**
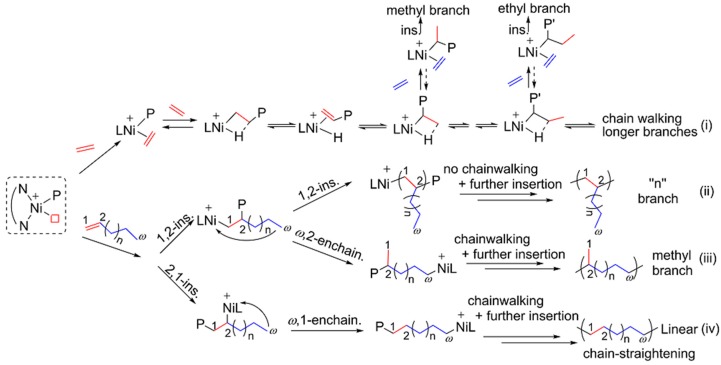
Mechanism for ethylene polymerization and 1-alkenes enchainment (L: NN = diimine Ligand; *ω-*position, where *ω* is the number of carbon atoms).

Highly branched polymers have been studied extensively in recent years. Compared to the corresponding linear polymer, highly branched polymers such as dendrimer and hyperbranched polymers possess unique physical properties stemming from their architectures [7]. One of the unique features of the diimine Ni(II) catalysts is the migration of the metal center along the growing polymer chain (chain-walking) so that the incoming next monomer unit is assembled onto the polymer backbone, giving branched polymers (Scheme 1). In 1-alkene polymerization with the Ni(II) catalysts, a large fraction of monomer inserts in a 2,1 manner. The following by chain-walking forms a 1, *ω-*enchainment (chain-straightening), resulting in a polyethylene sequence (Scheme 1iv). Wu *et al.* synthesized a camphyl α-diimine Ni(II) catalyst [13] which can polymerized ethylene, propylene and 1-alkenes in a living fashion under the optimized conditions. This catalyst exhibited high 1,3-enchainment fraction of 45% in propylene polymerization. Recently, Wang *et al.* reported synthesis of the polyethylene-based functionalized hyperbranched polymers using Pd(II) α-diimine catalyst [7].

It is well known that the steric and electronic effects of α-diimine ligands can affect the catalytic properties for olefin polymerization [14,15,16,17,18,19,20,21,22,23,24], especially the sterically bulky *ortho-*substituted α-diimine Ni(II) catalysts capable of generating high molecular weight polymers. For example, Long *et al*. reported that some sterically bulky α-diimine Ni(II) catalysts bearing a dibenzhydryl moiety were highly active in ethylene polymerization, generating polyethylene with high molecular weight [20]. Recently, Rhinehart *et al.* reported that a robust 2,6-bis(diphenylmethyl)-substituted Ni(II) α-diimine catalyst [14] was highly active and remarkably thermally stabile for ethylene polymerization. There are many papers for Ni(II) α-diimine complexes containing bulky substituents at 2,6-position on the aniline rings. However, the effect of *para-*position substituent of the phenyl groups on the catalytic behavior of α-diimine Ni(II) complexes and the property of resultant polymers has been reported only in a limited number of cases.

Recently, we reported that bulky chiral *ortho-sec-*diphenethyl-substituted [25,26] and *ortho-*phenyl substituted [27,28,29] α-diimine Ni(II) and Pd(II) complexes that exhibited high activities towards ethylene polymerization and produced highly-branched polyethylene upon activation with Et_2_AlCl. In this context, we have become very interested in phenyl-substituted α-diimine ligand Ni(II) precursors for polymerization of ethylene and 1-alkenes at the different conditions. In this work, we report the synthesis and application of six α-diimine Ni(II) complexes containing phenyl groups, and investigated the effects of the ligand on polymerization of ethylene and 1-alkene (1-hexene, 1-octene, 1-decene and 1-hexadecene) in detail.

## 2. Experimental Section

### 2.1. General Considerations and Materials

All manipulations were performed under nitrogen gas using standard Schlenk techniques. Research grade ethylene and propylene were purified by passing it through a deoxygenation and a dry columns. Methylene chloride and *o-*dichlorobenzene were pre-dried with 4 Å molecular sieves and distilled from CaH_2_ under dry nitrogen. Toluene, hexane, diethyl ether and 1,2-dimethoxyethane (DME) were distilled from sodium/benzophenone under nitrogen atmosphere and distilled before use. 1-Hexene, 1-octene, 1-decene and 1-hexadecene were purchased from Kanto Chemical Co., on Aldrich Chemical Company (Tokyo, Japan) were dried over CaH_2_, and distilled before use. Et_2_AlCl and MMAO were donated by Tosoh-Finechem. Pd(II) catalyst [30], **4** [30], **5** [30], **L4** [27], **L5** [29], **L6** [31], **C4** [27], **C5** [29] and **C7** [32] were prepared according to the literature procedures. Other chemicals were commercially obtained and purified with common procedures.

### 2.2. Instrumentation

^1^H and ^13^C NMR spectra of compounds were recorded on a Varian 500 MHz spectrometer (Varian, Inc., Palo Alto, CA, USA) and a Varian 400 instrument (Varian, Inc., Palo Alto, CA, USA) at ambient temperature using tetramethylsiane as an internal standard. ^1^H and ^13^C NMR of the polymer was measured by a Varian 500 MHz spectrometer at 130 °C using *o-*dichlorobenzene/1,1,2,2-tetrachoroethane-d_2_ (3:1) as a solvent or at room temperature using *o-*dichlorobenzene/chloroform-d (1:1). IR spectra were recorded on a JASCO FT/IR-300E spectrophotometer (Jasco, Corp., Tokyo, Japan) on KBr pellets. Elemental analysis was carried out using a Flash EA 1112 micro-analyzer (Thermo electron corporation, Waltham, MA, USA). The molecular weight of the polymer was determined by a Tosoh HLC-8320GPC chromatograph (Tosoh Asia Pte. Ltd., Tokyo, Japan) at 40 °C using tetrahyrdofuran (THF) as an eluent, calibrated with polystyrene standard. DSC analysis of the polymer was performed by a SII EXSTAR6000 system (Hitachi, Tokyo, Japan).

### 2.3. Synthesis and Characterizations

#### 2.3.1. Synthesis of Aniline Derivatives **1**–**3**

##### Synthesis of 2,6-Dimethyl-4-Phenylaniline **1**

4-Bromo-2,6-dimethylaniline (0.40 g, 2.00 mmol), phenylboronic acid (0.26 g, 2.10 mmol), K_2_CO_3_ (0.55 g, 4.00 mmol) and **Pd-Cat.** (0.026 g, 0.040 mmol), were placed in a 100-mL flask and allowed to stir at room temperature for 12 h, in the presence of PEG-400/H_2_O (30 mL/1.0 mL). The mixture was extracted three times with 10 mL diethyl ether. The combined organic phase was dried over MgSO_4_, filtered, and the solvent was removed. The residue was purified by chromatography on silica gel with petroleum ether/ethyl acetate (*v*/*v* = 25:1) to give 2,6-dimethyl-4-phenylaniline **1** (0.34 g, 86% yield). ^1^H NMR (400 MHz, CDCl_3_, ppm): *δ* 7.45–7.50 (m, 2H, aryl–*H*), 7.28–7.35 (m, 2H, aryl–*H*), 7.13–7.21 (m, 3H, aryl–*H*), 3.54 (s, 2H, –N*H_2_*), 2.15 (s, 6H, –C*H*_3_). ^13^C NMR (100 MHz, CDCl_3_, ppm): *δ* 142.22, 141.41, 130.90, 128.50, 126.93, 126.42, 125.98, 121.90, 17.73 (–*C*H_3_).

##### Synthesis of 2,4-Dimethyl-6-Phenylaniline **2**

Using 2-bromo-4,6-dimethylaniline (0.40 g, 2.00 mmol) and phenylboronic acid (0.26 g, 2.10 mmol), and applying the same synthetic procedure for **1**, **2** was obtained as oily liquid (0.29 g, 74% yield). ^1^H NMR (400 MHz, CDCl_3_, ppm): *δ* 7.42–7.43 (m, 4H, aryl–*H*), 7.30–7.34 (m, 1H, aryl–*H*), 6.89 (s, 1H, aryl–*H*), 6.83 (s, 1H, aryl–*H*), 3.51 (s, 2H, –N*H_2_*), 2.25 (s, 3H, –C*H*_3_), 2.19 (s, 3H, –C*H*_3_). ^13^C NMR (100 MHz, CDCl_3_, ppm): *δ* 139.92, 138.97, 130.35, 129.17, 128.69, 128.65, 127.61, 127.21, 126.97, 122.65, 20.34 (–*C*H_3_), 17.47 (–*C*H_3_).

##### Synthesis of 2-Methyl-4,6-Diphenylaniline **3**

Using 2,4-dibromo-6-methylaniline (0.53 g, 2.00 mmol) and phenylboronic acid (0.52 g, 4.20 mmol), and applying the same synthetic procedure for **1**, **3** was obtained as oily liquid (0.39 g, 75% yield). ^1^H NMR (400 MHz, CDCl_3_, ppm): *δ* 7.50 (d, *J* = 8.4 Hz, 2H, aryl–*H*), 7.37–7.44 (m, 4H, aryl–*H*), 7.27–7.33 (m, 4H, aryl–*H*), 7.16–7.21 (m, 2H, aryl–*H*), 3.69 (s, 2H, –N*H_2_*), 2.22 (s, 3H, –C*H*_3_). ^13^C NMR (100 MHz, CDCl_3_, ppm): *δ* 141.17, 141.12, 139.71, 131.02, 129.23, 128.85, 128.59, 128.34, 127.76, 127.24, 126.94, 126.43, 126.18, 122.77, 18.09 (–*C*H_3_).

#### 2.3.2. Synthesis of Ligands **L1**–**L3**

##### Synthesis of bis[*N*,*N*′-(2,6-Dimethyl-4-Phenylphenyl)imino]Acenaphthene **L1**

Formic acid (0.20 mL) was added to a stirred solution of 1,2-acenaphthylenedione (0.091 g, 0.50 mmol) and **1** (0.22 g, 1.10 mmol) in MeOH (30 mL). The mixture was refluxed for 12 h, then cooled and the precipitate was separated by filtration. The solid was recrystallized from MeOH/CH_2_Cl_2_ (*v*/*v* = 15:1), washed with cold ethanol and dried under vacuum (0.22 g, 80% yield). ^1^H NMR (400 MHz, CDCl_3_, ppm): *δ* 7.92 (d, *J* = 8.0 Hz, 2H, naphtha–*H*), 7.73 (m, 4H, naphtha–*H*), 7.41–7.49 (m, 8H, aryl–*H*), 7.26 (s, 4H, aryl–*H*), 6.87 (d, *J* = 7.2 Hz, 2H, aryl–*H*), 2.22 (s, 12H, –C*H*_3_). ^13^C NMR (100 MHz, CDCl_3_, ppm): *δ* 160.99 (*C*=N), 148.64, 140.97, 140.65 (naphtha–*C*), 136.35, 131.05 (naphtha–*C*), 129.58, 129.02 (naphtha–*C*), 128.69, 128.32 (naphtha–*C*), 126.93, 126.79, 126.71, 125.28 (naphtha–*C*), 122.64 (naphtha–*C*), 17.99 (–*C*H_3_). Anal. Calcd. for C_40_H_32_N_2_: C, 88.85; H, 5.97; N, 5.18. Found: C, 88.91; H, 6.02; N, 5.14. FT-IR (KBr): 1640 cm^−1^ (*v*_C=N_).

##### Synthesis of bis[*N*,*N*′-(2,4-Dimethyl-6-Phenylphenyl)imino]Acenaphthene **L2**

Using the same synthetic procedure for **L1**, **L2** was obtained as an orange-red powder (0.20 g, 74% yield). ^1^H NMR (400 MHz, CDCl_3_, ppm): *δ* 7.72 (d, *J* = 8.4 Hz, 2H, naphtha–*H*), 7.45 (m, 4H, naphtha–*H*), 7.26–7.35 (m, 2H, aryl–*H*), 7.07–7.12 (m, 8H, aryl–*H*), 7.00 (d, *J* = 6.8 Hz, 2H, aryl–*H*), 7.69 (d, *J* = 7.2 Hz, 2H, aryl–*H*), 2.44 (s, 6H, –C*H*_3_), 2.07 (s, 6H, –C*H*_3_). ^13^C NMR (100 MHz, CDCl_3_, ppm): *δ* 160.43 (*C*=N), 145.73, 140.49, 139.65 (naphtha–*C*), 133.44, 130.65 (naphtha–*C*), 129.80, 129.58, 129.35 (naphtha–*C*), 128.66, 128.46 (naphtha–*C*), 127.88, 127.72, 127.41, 126.25, 125.94 (naphtha–*C*), 122.47 (naphtha–*C*), 20.98 (–*C*H_3_), 17.99 (–*C*H_3_). Anal. Calcd. for C_40_H_32_N_2_: C, 88.85; H, 5.97; N, 5.18. Found: C, 88.95; H, 6.00; N, 5.11. FT-IR (KBr): 1641 cm^−1^ (*v*_C=N_).

##### Synthesis of bis[*N*,*N*′-(2-Methyl-4,6-Diphenylphenyl)imino]Acenaphthene **L3**

Using the same synthetic procedure for **L1**, **L3** was obtained as orange-red powder (0.36 g, 70% yield). ^1^H NMR (400 MHz, CDCl_3_, ppm): *δ* 7.96–8.01 (m, 4H, naphtha–*H*), 7.86 (d, *J* = 8.0 Hz, 2H, naphtha–*H*), 7.60–7.67 (m, 4H, aryl–*H*), 7.51 (s, 2H, aryl–*H*), 7.38–7.42 (m, 4H, aryl–*H*), 7.29–7.35 (m, 6H, aryl–*H*), 7.67 (t, *J* = 7.2 Hz, 4H, aryl–*H*), 7.96–7.00 (m, 2H, aryl–*H*), 7.83 (d, *J* = 7.2 Hz, 2H, aryl–*H*), 2.12 (s, 6H, –C*H*_3_). ^13^C NMR (100 MHz, CDCl_3_, ppm): *δ* 160.03 (*C*=N), 146.51, 142.89, 140.37 (naphtha–*C*), 139.19, 137.57, 131.95 (naphtha–*C*), 130.79 (naphtha–*C*), 130.57, 129.32 (naphtha–*C*), 129.14, 128.83, 128.48, 128.20, 127.87, 127.22, 127.01, 126.80, 126.26 (naphtha–*C*), 122.71, 122.02 (naphtha–*C*), 18.36 (–*C*H_3_). Anal. Calcd. for C_50_H_36_N_2_: C, 90.33; H, 5.46; N, 4.21. Found: C, 90.29; H, 5.51; N, 4.26. FT-IR (KBr): 1639 cm^−1^ (*v*_C=N_).

#### 2.3.3. Synthesis of Complexes **C1**–**C3** and **C6**

##### Synthesis of {bis[*N*,*N*′-(2,6-Dimethyl-4-Phenylphenyl)imino]Acenaphthene}Dibromonickel **C1**

[(DME)NiBr_2_] (0.12 g, 0.40 mmol) and **L1** (0.22 g, 0.40 mmol) were combined in a Schlenk flask under a N_2_ atmosphere. CH_2_Cl_2_ (20 mL) was added, and the reaction mixture was stirred at room temperature for 24 h. The resulting suspension was filtered. The solvent was removed under vacuum and the residue was washed with diethyl ether (3 × 15 mL), and then dried under vacuum at room temperature to give Ni(II) complex **C1** (0.26 g, 86% yield). Anal. Calcd. for C_40_H_32_Br_2_N_2_Ni: C, 63.28; H, 4.25; N, 3.69. Found: C, 63.34; H, 4.30; N, 3.75. FT-IR (KBr): 1639 cm^−1^ (*v*_C=N_). Single crystals of complex **C1** suitable for X-ray analysis were obtained at room temperature by dissolving the nickel complex in CH_2_Cl_2_, following by slow layering of the resulting solution with *n-*hexane.

##### Synthesis of {bis[*N*,*N*′-(2,4-Dimethyl-6-Phenylphenyl)imino]Acenaphthene}Dibromonickel **C2**

Using the same synthetic procedure for **C1**, **C2** was obtained as a dark-red powder (0.28 g, 88% yield). Anal. Calcd. for C_40_H_32_Br_2_N_2_Ni: C, 63.28; H, 4.25; N, 3.69. Found: C, 63.31; H, 4.28; N, 3.76. FT-IR (KBr): 1635 cm^−1^ (*v*_C=N_).

##### Synthesis of {bis[*N*,*N*′-(2-Methyl-4,6-Diphenylphenyl)imino]Acenaphthene}Dibromonickel **C3**

Using the same synthetic procedure for **C1**, **C3** was obtained as a dark-red powder (0.32 g, 90% yield). Anal. Calcd. for C_50_H_36_Br_2_N_2_Ni: C, 67.98; H, 4.11; N, 3.17. Found: C, 68.05; H, 4.07; N, 3.13. FT-IR (KBr): 1638 cm^−1^ (*v*_C=N_).

##### Synthesis of {bis[*N*,*N*′-(2,4,6-Triphenylphenyl)imino]Acenaphthene}Dibromonickel **C6**

Using the same synthetic procedure for **C1**, **C6** was obtained as a dark-red powder (0.35 g, 87% yield). Anal. Calcd. for C_60_H_40_Br_2_N_2_Ni: C, 71.53; H, 4.00; N, 2.78. Found: C, 71.59; H, 3.96; N, 2.81. FT-IR (KBr): 1637 cm^−1^ (*v*_C=N_).

### 2.4. X-ray Structure Determinations

Single crystals of complex **C1** suitable for X-ray analysis was obtained by dissolving the Ni(II) complex in CH_2_Cl_2_, followed by slow layering of the resulting solution with *n-*hexane. Data collections were performed at 296(2) K on a Bruker SMART APEX diffractometer with a CCD area detector, using graphite monochromated Mo*K*α radiation (*λ* = 0.71073 Å). The determination of crystal class and unit cell parameters was carried out by the SMART program package. The raw frame data were processed using SAINT and SADABS to yield the reflection data file. The structures were solved by using the SHELXTL program. Refinement was performed on *F*^2^ anisotropically for all non-hydrogen atoms by the full-matrix least-squares method. The hydrogen atoms were placed at the calculated positions and were included in the structure calculation without further refinement of the parameters. Crystal data, data collection, and refinement parameters are listed in Table S1 (Supplementary Materials).

### 2.5. Ethylene Polymerization

Ethylene polymerization was performed in a 100-mL glass reactor equipped with a magnetic stirrer. After drying the reactor under N_2_ atmosphere, toluene was added to the reactor. The solvent was then saturated with a prescribed ethylene pressure (1.2 atm). The co-catalyst (Et_2_AlCl or MMAO) was added in Al/Ni molar ratios in the range of 200–1000 to the reactor via a syringe, the solution was thermostated to the desired temperature and allowed to equilibrate for 10 min. Then, the catalyst solution in toluene was added to the reactor. The polymerization, conducted under 1.2 atm of ethylene pressure, was terminated with 200 mL of a 3% HCl–MeOH solution. The polymers obtained were adequately washed with methanol and dried under vacuum at 60 °C for 6 h.

### 2.6. 1-Alkenes Polymerization

Polymerization of 1-alkene (1-hexene, 1-octene, 1-decene and 1-hexadecene) was carried out in a 100-mL glass reactor equipped with a magnetic stirrer. After drying the reactor under N_2_ atmosphere, toluene was added to the reactor. 1-Alkene was added to the toluene kept at polymerization temperature via a syringe. Then the co-catalyst MMAO was added to the toluene and the mixture was stirred for 10 min. Polymerization was started by the addition of the catalyst solution. Polymerization was terminated with 200 mL of a 3% HCl–MeOH solution. The polymers obtained were adequately washed with methanol and dried under vacuum at 60 °C for 6 h.

## 3. Results and Discussion

### 3.1. Synthesis and Characterization of the Organic Compounds and Their Complexes **C1**–**C7**

**Scheme 2:**
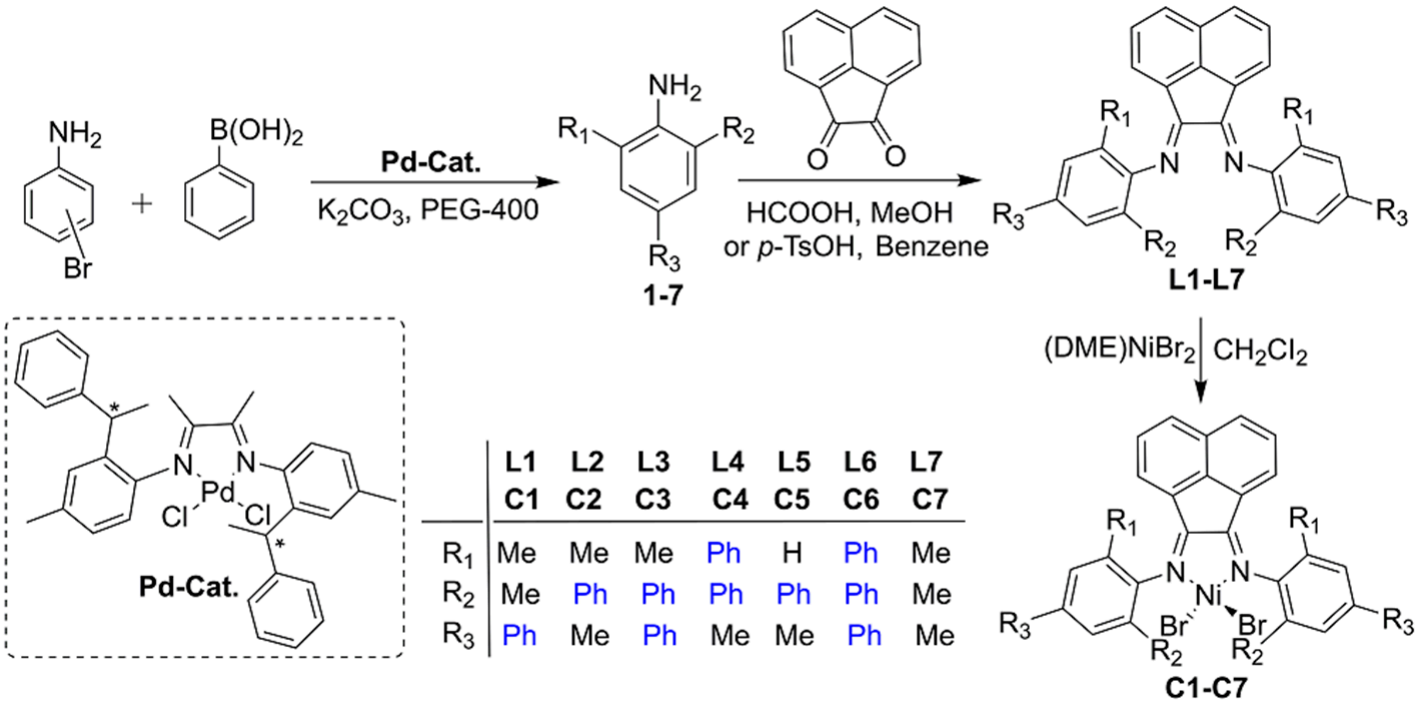
Synthesis of α-diimine ligands **L1**–**L7** and their Ni(II) complexes** C1**–**C7**.

The synthesis of ligands **L1**–**L7** and their complexes **C1**–**C7** are outlined in Scheme 2. After the protection of the amino group by acetic acid, the aniline derivatives were brominated. The Suzuki coupling reaction of the bromoaniline derivatives and phenylboronic acid catalyzed by a Pd(II) catalyst (**Pd-Cat.**) in PEG-400/H_2_O led to the corresponding phenyl-substituted aniline derivatives **1**–**6**. The ligands **L1**–**L7** were prepared by the condensation of two equivalents of the appropriate aniline with one equivalent of acenaphthequinone, in the presence of formic acid or *p-*toluenesulfonic acid, as a catalyst. Compounds **L1**–**L7** were well characterized by IR, ^1^H NMR, ^13^C NMR spectroscopy and elemental analysis.

The reaction of equimolar amounts of NiBr_2_(DME) and the α-diimine ligands **L1**–**L7** in CH_2_Cl_2_ led to the displacement of 1,2-dimethoxyethane and afforded the Ni(II) complexes **C1**–**C7** as a moderately air-stable deep red microcrystalline solid in good yields. These complexes were characterized by IR spectroscopy and elemental analysis. The elemental analysis of complex **C1** fitted the molecular structure obtained by the X-ray structural studies (see below).

### 3.2. X-ray Crystallographic Studies

**Figure 1:**
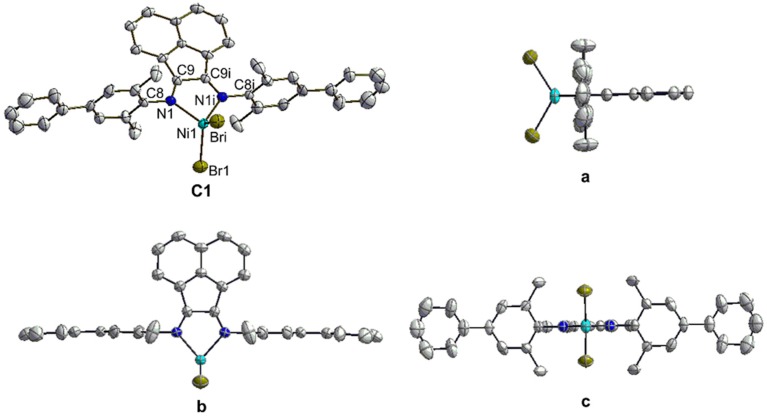
Molecular structures of the catalyst precursor **C1** with 50% probability level, and H atoms have been omitted for clarity. Selected bond lengths (Å) and angles (deg) for complexes: Br1–Ni1, 2.3343(12); Ni1–N1, 2.041(5); N1–C9, 1.290(9); N1–C8, 1.427(8); Br1–Ni1–Br1i, 117.34(8); N1–Ni1–Br1i, 112.96(6); C9–N1–C8, 118.0(6).

The molecular structures of complex **C1** was confirmed by single-crystal X-ray diffraction and the corresponding ORTEP diagram is shown in Figure 1. Crystal data, data collection and refinement parameters are listed in Table S1 (Supplementary Materials). The coordination geometry around the metal center is tetrahedral for the Ni(II) complex **C1**. The molecular structure of the Ni(II) complex **C5** was reported in the light of our recent publication (Figure S1) [29], and **C7** was also reported in the literature [32].

The molecular diagram with selected bond distances and angles for complex **C1** are shown in Figure 1. The X-ray structure analysis indicates that complex **C1** has a pseudo-tetrahedral geometry about the Ni(II) center, showing *C*_2v_ molecular symmetry. The two methyl in the 2,6-position of the benzenamine fragments in **C1** located above and below the Ni1 coordinative geometry plane (Figure 1a). The aryl rings of each of the α-diimines lie nearly perpendicular to the plane formed by the nickel and coordinated nitrogen atoms, and face each other in a staggered vertical conformation (Figure 1b,c). The two imino C=N bonds have typical double bond character with C9=N1 bond lengths of 1.290 Å. Its structure is similar to those reported in the literature for a [NiBr_2_(α-diimine)] compound characterized by X-ray diffraction, {bis[*N*,*N′-*(2,4,6-trimethylphenyl)imino]acenaphthene}dibromonickel **C7** [32]. In fact, the Ni–N bond distances in complex **C1** (2.041 Å) are similar to those determined for the complex **C7** (2.021 Å), and the Ni–Br bond distances in complexes **C1** and **C7** are almost identical (2.3343 Å for complex **C1** *vs.* 2.323 Å for complex **C7**). In addition, the N–Ni–Br angles (112.96° for complex **C1**) are also approximate to those for complex **C7** (114.4°).

### 3.3. Ethylene Polymerization

Effects of co-catalyst and polymerization conditions on ethylene polymerization were initially explored using complex **C1**. At 20 °C, the influence of the [Al]/[Ni] with Et_2_AlCl was investigated by increasing the [Al]/[Ni] molar ratio from 200 to 1000 (entries 1−5, Table 1). The activity of complex **C1** did not significantly depend on the [Al]/[Ni] ratio and the highest activity of 2.08 × 10^6^ g PE/(mol Ni h) was achieved with the [Al]/[Ni] ratio of 600 (entry 3, Table 1). In comparison **C1-**MMAO system exhibited higher activity than the **C1-**MMAO system (entries 3 and 11, Table 1).

Then, the influence of the polymerization temperature was studied by varying the temperature from 0 to 80 °C (entries 3 and 6−14, Table 1) at the [Al]/[Ni] molar ratio of 600 for 10 min with the Et_2_AlCl and the MMAO systems. The observed activities decreased as the reaction temperature increased from 0 to 80 °C. It is worth mentioning, however, that the polymerization at 80 °C still maintained good activity, 1.03 × 10^6^ g PE/(mol Ni h) in the case of MMAO (entry 14, Table 1). The lower activities at higher temperatures should be ascribed to the partial deactivation of the active species and the lower solubility of ethylene in toluene at elevated temperatures [6].

Along with increasing the Al/Ni ratio (entries 1–5, Table 1), there have been no significant changes in the polymer molecular weight trend. On the other hand, the polymer molecular weight was monotonously decreased with elevating the temperature with narrow molecular weight distributions of *M*_w_/*M*_n_ < 2. The maximum molecular weight was observed at 0 °C (entries 6 and 10, Table 1), while the higher temperature produced the polyethylene with a lower molecular weight (entries 8, 9, 13 and 14, Table 1). This means that fast chain transfer take place at higher temperatures. In general, compared with the systems using MMAO, the resulting polyethylenes with Et_2_AlCl showed somewhat lower molecular weights.

The polymerization of ethylene with α-diimine Ni(II) complexes typically provides highly branched amorphous polyethylenes through a chain-walking mechanism (Scheme 1i). Polymer branching can be controlled by variation of temperature, and increases dramatically with temperature. As shown in Table 1, **C1**-Et_2_AlCl produced polyethylene with branching numbers/1000 C of 105 at 20 °C, 119 at 40 °C, 134 at 60 °C and 140 at 80 °C (entries 3 and 7–9). A similar trend was observed with **C1**-MMAO, PE with branching numbers/1000 C of 121 at 20 °C, 133 at 40 °C, 139 at 60 °C and 145 at 80 °C (entries 11–14). The branching numbers of 60 and 80 °C rival those obtained with palladium catalysts [3,4].

It is known that the catalytic activity and the molecular weight of the produced polymers have a close correlation with the steric and electronic characteristics of the Ni(II) α-diimine complexes [24]. All the Ni(II) pre-catalysts **C1**–**C7** were applied to ethylene polymerization activated by MMAO at 20 °C under 1.2 atm of ethylene (entries 11 and 15–20, Table 1).

Five α-diimine Ni(II) **C2**–**C6**/MMAO catalytic systems are highly active for ethylene polymerization due to the bulky *ortho-*phenyl substituents on the aryl rings of α-diimine Ni(II) complexes [**C6**-MMAO, the highest catalytic activity: 3.12 × 10^6^ g PE/(mol Ni h)] (entry 19, Table 1). Complex **C1** bearing a phenyl group in the *para-*aryl position, activated by MMAO, exhibited higher activity toward ethylene polymerization than the corresponding methyl substituted complex **C7** [activity: 2.17 × 10^6^ g PE/(mol Ni h); at 20 °C] (entries 11 and 20, Table 1), which may was attributed to the ability of the conjugation effect phenyl substituent in the *para-*aryl position. As a result, the activity was found to decrease in the following order: **C6** ≥ **C4** ≥ **C3** ≥ **C2** ≥ **C1** ≥ **C5** > **C7**. The catalytic performances could not be explained with the sole factor of either steric or electronic influences, but likely were caused by a combination of both steric and electronic effects [6].

According to the GPC measurement, all obtained polyethylenes generally showed a high molecular weight (*M*_n_ > 10^5^ g/mol) and a narrow polydispersity at room temperature (entries 11 and 15–20, Table 1), and the molecular weight decreased slightly in the order: **C6** ≥ **C4** ≥ **C3** ≥ **C2** ≥ **C1** ≥ **C5** > **C7**. These results indicated that the rate of chain propagation was greatly promoted by the *para-* and *ortho-*phenyl groups of the ligand’s aryl rings.

The performance of the α-diimine Ni(II) complexes are significantly affected by position phenyl-substituents on the aniline rings. Highly branched polyethylenes can be produced at lower ethylene pressures and higher temperatures with Ni(II) catalyst systems [19]. The chain branching degree of the polyethylenes produced was determined by ^1^H NMR spectroscopy. As shown in Table 1, the catalyst systems **C1**-Et_2_AlCl and **C1**-MMAO the branching numbers are 105 and 121, respectively (entries 3 and 11, Table 1). These numbers are substantially higher than those for **C7** (entry 19, Table 1) and related systems [31]. The number of chain branching was also depended on the pre-catalysts and decreased as follows: **C6** (125) ≥ **C3** (124) ≥ **C1** (121) > **C4** (106) ≥ **C2** (103) ≥ **C5** (94) > **C7** (81). The results indicated that the *para*-phenyl substituted α-diimine Ni(II) complexes (**C6**, **C3** and **C1**) afford polyethylenes with a considerably higher branching degree than the corresponding *para*-methyl substituted complexes (**C4**, **C2**, **C5** and **C7**), which may was attributed to the conjugation ability of the phenyl substituent in the *para-*aryl position.

We recorded the ^13^C NMR spectra of the polymers obtained at 0–80 °C using **C1** to evaluate the microstructure and properties of the polymers in detail, and the results are summarized in Table 2. The spectra obtained were analyzed according to the literature [22,23]. We have previously reported that the polyethylene produced by an chiral naphthyl-α-diimine Ni(II)-Et_2_AlCl at 40 °C possessed short chain branches (such as methyl, ethyl, propyl and butyl) and long chain branches [26].

The polymerization of ethylene with phenyl-substituted α-diimine Ni(II) complex **C1** typically provides highly branched amorphous polyethylenes through a chain-walking mechanism. For example, the polyethylene produced at 20 °C possessed 122 branches/1000 C including 88 methyl, 9 ethyl, 3 propyl, 3 butyl, 2 *sec-*butyl and 17 longer chains (entry 11, Table 2), while the polyethylene obtained at 80 °C (entry 14, Table 2) had 146 branches/1000 C, containing 88 methyl, 16 ethyl, 2 propyl, 8 butyl, 10 *sec-*butyl and 22 longer chains. It was also found that polyethylene with 105 branches/1000 C (including 74 methyl, 7 ethyl, 5 propyl, 5 butyl and 14 longer chains) were obtained at 20 °C by **C1**-Et_2_AlCl (entry 3, Table 2). The rationale for the formation of the major types of branches (methyl, ethyl, propyl, butyl and longer chains) in the polyethylenes obtained in this work is shown in Scheme 1i.

The DSC traces for these highly branched polyethylenes were exhibited in the Table 2 (Figure S47). The results show no melting points (*T*_m_), indicating that the obtained polymers are amorphous elastomers due to highly branched structure. Similar to classic nickel α-diimine catalysts, the glass transition temperatures (T_g_) of the polymers are low in the range from −56.7 to −68.2 °C, *T*_g_ decreased with increasing temperature (entries 10–14, Table 1). The increase in branching with an increase in temperature results from an increase in the rate of chain-walking relative to the rate of insertion [13].

**Figure 2:**
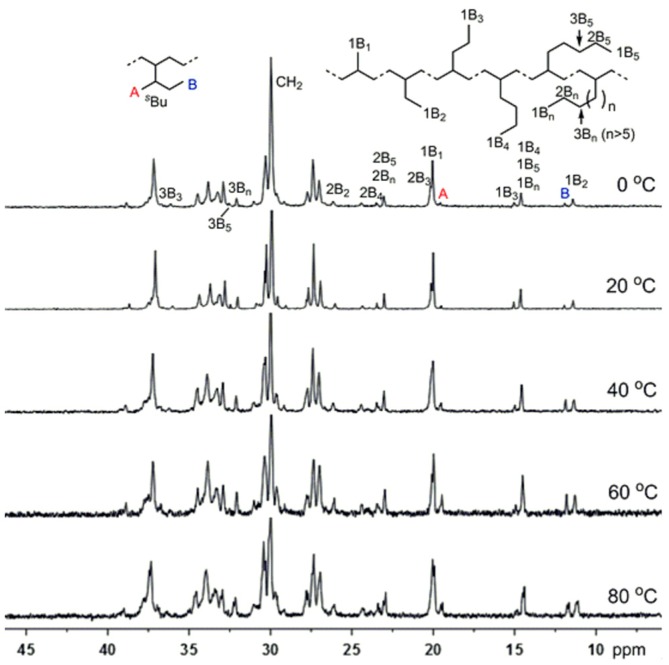
^13^C NMR spectrum of polyethylenes obtained with **C1**-MMAO at 0–80 °C (A and B refer to methyl carbon of *sec-*butyl branches, entries 10–14, Table 1).

The ^13^C NMR analysis of the polyethylenes revealed the presence of a hyperbranched (branch on branch) moiety, specifically a *sec-*butyl branch (Table 2 and Figure 2). In the ^13^C NMR spectrum of the polyethylene reported resonances at 12.17 and 19.87 ppm confirm the formation of *sec-*butyl branches [12]. As shown in Table 2 and Figure 2, the density of *sec-*butyl branches was dependent on reaction temperature and cocatalyst. Table 2 indicated that the formation of *sec-*butyl branches produced by **C1**-Et_2_AlCl was more suppressed than **C1**-MMAO (entries 3, 7, 11 and 12, Table 2). Although the polyethylene branching increased dramatically with temperature, the density of each branchof polyethylenes has a different changing trend agaist polymerization temperature. The number of *sec-*butyl branches determined by ^13^C NMR increased as the polymerization temperature raised in the range from 0 to 60 °C (entries 10–13, Table 2), the maximum being reached at 60 °C (entry 13), and then the number of *sec-*butyl branches decreased slightly with an increase temperature (entries 13 and 14). Besides, the number of ethyl branches increased slightly with an increase temperature (entries 10−14, Table 2), while the influence of polymerization temperature on the number of methyl and propyl branches were not observed.

### 3.4. Higher 1-Alkenes Polymerization

Higher 1-alkene polymerizations (1-hexene, 1-octene, 1-decene and 1-hexadecene) were performed using complex **C1** with the [Al]/[Ni] molar ratio of 300 for 30 min with MMAO as a cocatalyst. The results listed in Table 3 show that the catalytic system exhibited good activity for higher 1-alkene polymerization to produce polymers with high molecular weight and narrow molecular weight distribution (*M*_w_/*M*_n_ = 1.04–1.41), indicating the living characteristics of the polymerizations.

The catalytic activity and polymer molecular weight in the polymerization have a close correlation with the length of monomer, monomer concentration and reaction temperature [4,13]. When the high monomer concentration of 1.5 M, **C1**-MMAO displayed higher activity toward the 1-alkene polymerization to produce polymers with high molecular weight [activity: 126 ~ 238 kg polymer/(mol Ni h); *M*_n_: 1.52 ~ 3.12 × 10^5^ g/mol] in comparison with the low monomer concentration of 0.3 M (entries 1−5, 8, 12 and 14, Table 3). However, **C1**-MMAO afforded polymers with very narrow molecular weight distribution (*M*_w_/*M*_n_ = 1.04–1.09) with relatively low monomer concentration of 0.3 M, indicating the living characteristics of the polymerizations. As the length of the monomer increases, the catalytic activity and polymer molecular weight increased with relatively high monomer concentration of 1.5 M. However, catalytic activity increased, and then decreased with the low monomer concentration of 0.3 M. For example, the polymerization of 1-hexadecene was conducted by **C1**-MMAO with the low monomer concentration of 0.3 M, displayed relatively lower activity and obtained polymer with high molecular weight (entries 13 and 14, Table 3).

The 1-decene polymerization was conducted by **C1**-MMAO at temperatures from −20 to 80 °C with the low monomer concentration of 0.3 M (entries 6–11, Table 3). With an increase in the polymerization temperature, the maximum activity was observed at 20 °C (entry 8, Table 3), and the maximum *M*_n_ was observed at 40 °C (entry 9, Table 3). In addition, the higher temperature resulted in a lower activity and produced the poly(1-decene) with a lower molecular weight (entries 10 and 11, Table 3). This means that both the deactivation of active species and fast chain transfer take place at elevated temperatures [4], which was consistent with the analogous pre-catalysts [13]. The narrow polydispersity (*M*_w_/*M*_n_ = 1.09–1.41) was observed for all poly(1-decene)s obtained, and *M*_w_/*M*_n_ value gradually increased by raising the temperatures from 0 to 80 °C.

**Figure 3:**
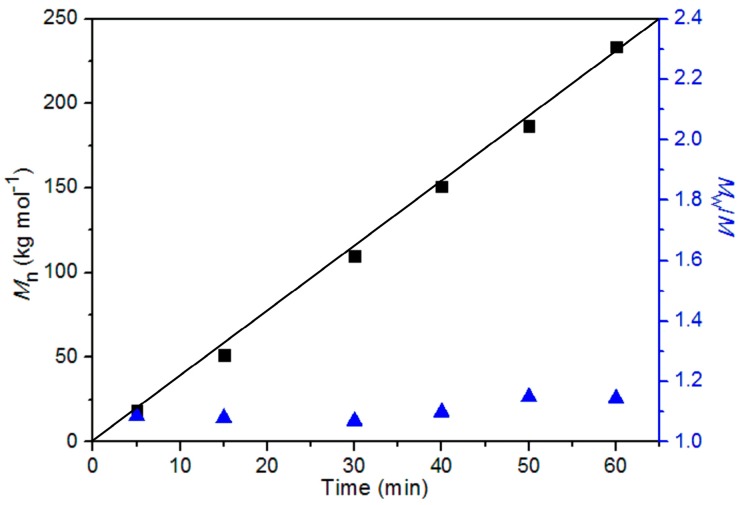
Plots of *M*_n_ (■) and *M*_w_/*M*_n_ (▲) as a function of reaction time for the polymerization of 1-decene (20 °C, Table S3).

Polymerization of 1-decene was investigated with **C1**-MMAO at 20 °C with the [Al]/[Ni] molar ratio of 300, and the results are summarized in Table S3. Figure S11 shows the GPC curves of the polymers obtained at different polymerization time, which shifts to the higher molecular weight region with the increasing reaction time. Figure 3 shows the plots of *M*_n_ and *M*_w_/*M*_n_ as a function of polymerization time. The plot of *M*_n_ against polymerization time shows a good linear relationship accompanied by a very narrow molecular weight distribution (*M*_w_/*M*_n_ = 1.07−1.11), and the *N* value was almost constant during the polymerization (Table 3, see Supplementary Materials). The *M*_n_ value also increased linearly against the conversion (Figure S10), and the *M*_w_/*M*_n_ value was kept narrow during the polymerization. This result verified that the 1-decene polymerization with **C1**-MMAO proceeded in a living manner at 20 °C within a certain period of time. For early transition metal catalysts, living polymerization of 1-alkene at room temperature has been achieved only in rare instances.

The total branching level was calculated by ^1^H NMR spectroscopy. The branching degree of the poly(1-alkene) decreased with increasing chain length of the monomer: 1-hexene (142) > 1-octene (107) > 1-decene (93) > 1-hexadecene (50) (20 °C, entries 1, 3, 5 and 12, Table 3). The total branching degree of the obtained poly(1-alkene)s are always less branched than the theoretical value (1-hexene, 167/1000 C; 1-octene, 125/1000 C; 1-decene, 100/1000 C; 1-hexadecene, 63/1000 C). The deviation from the chain branching of regioregular polymers indicates that the 2,1-insertion of 1-alkene always results in *ω*,1-enchainment (Scheme 1). The monomer concentration determines the total branching number of the obtained polymer. An observed tendency is that increasing the monomer concentration results in a reducing total branching. For example, it drops from 142 to 121 branches/1000 C, 107 to 97 branches/1000 C, 93 to 85 branches/1000 C and from 50 to 46 branches/1000 C for poly(1-hexene), poly(1-octene), poly(1-decene) and poly(1-hexadecene), respectively (1.5 or 0.3 mol/L, entries 1–5, 8, 12 and 14, Table 3). These branching numbers were higher than those of the previously reported ones under the similar conditions [3,4,10,13]. For example, Leone *et al.* reported that a typical α-diimine Ni(II) catalyst gave poly(1-hexene) with77–92 branches/1000 C and poly(1-octene) with 65–74 branches/1000 C at 20 °C, which were independent of the monomer concentration (0.2–2.5 mol/L) [10]. The branching degree of the obtained poly(1-decene)s decreased with the increasing temperature (entries 6−11, Table 3), which results from chain-walking.

All synthesized poly(1-alkene)s were characterized by ^13^C NMR for quantification of the total content of methyl groups and branching distribution on the basis of previous work [15]. The results are summarized in Table 3.

**Figure 4:**
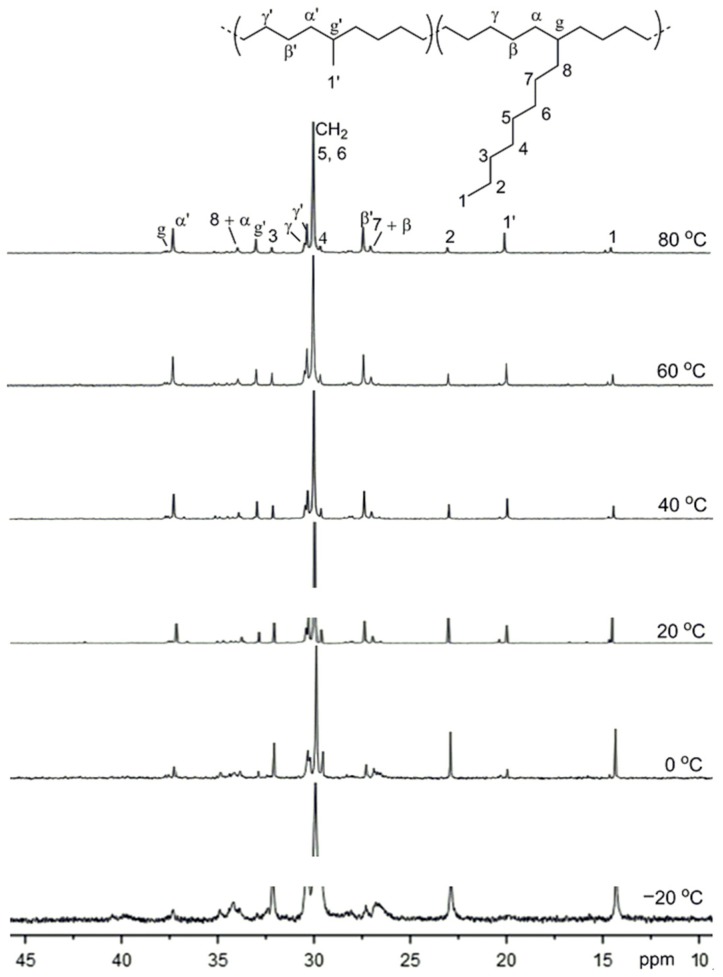
^13^C NMR spectra of poly(1-decene)s obtained with **C1**-MMAO at −20–80 °C (entries 6–10, Table 3).

**Figure 5:**
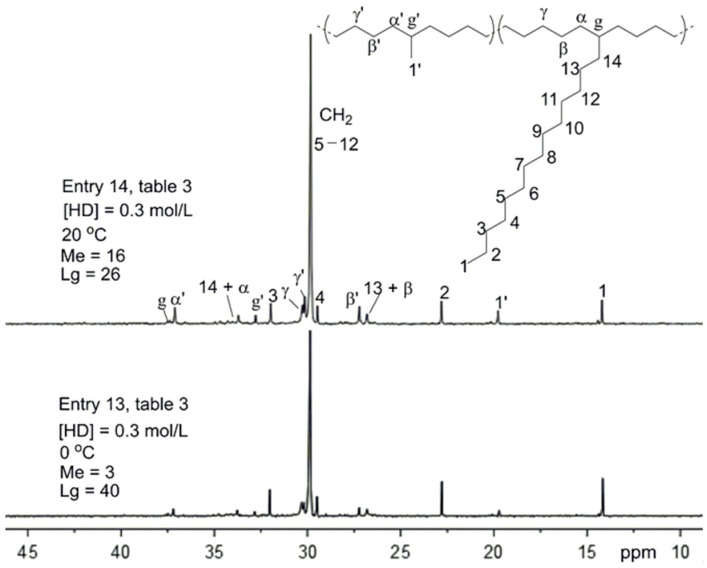
^13^C NMR spectra of poly(1-hexadecene)s obtained with **C1**-MMAO at 0 and 20 °C (entries 13 and 14, Table 3).

Poly(1-hexene)s show three major branch lengths (*i.e.*, methyl, butyl and longer than hexyl branches) branch (Figures S22 and S23) while 1-octene (Figures S24 and S25), 1-decene (Figure 4) and 1-hexadecene (Figure 5) polymers have almost exclusively methyl and long branches, because these monomers give hexyl, octyl and tetradecyl branches respectively in 1,2-insertion (Scheme 1ii).

The long branches of poly(1-decene)s determined by ^13^C NMR decreased as the polymerization temperature raised in the range from −20 to 80 °C (Figure 4), while the number of methyl branches increased (entries 6−11, Table 3). The results indicated the chain-walking depended on the polymerization temperature, and the 10,1- and 10,2-enchainements increased with increasing the polymerization temperature (Scheme 1). Besides, the small amount of propyl branches did not significantly depend on the temperature.

The ^13^C NMR spectra of two poly(1-hexadecene)s obtained at 0 and 20 °C are also shown in Figure 5. The intense signal of methylene −CH_2_− sequences and low intense signals due to methyl and longer branches were detected. No carbons of adjacent methyl branches (14.5–17.5 ppm) and ethyl branch were observed in the spectra. This indicates that the insertion of 1-hexadecene into a secondary Ni−alkyl bond did not occur [2,10]. Therefore, the 2,1-insertion of 1-hexadecene always evolves into a 16,1-enchainement to give long methylene sequences (Scheme 1iv). Besides, the reaction temperature significantly determines the branch-type distribution. The predominance of longer branches at lower temperature (0 °C) means that the predominate 1,2-insertion followed by successive monomer insertion before isomerization (Scheme 1ii). The content of *ω*,1-enchainments slightly decreased with the length of the monomer increased (Scheme 1).

The monomer concentration also significantly affects the branch-type distribution, and the ratio of methyl and longer branches can be tuned by changing the monomer concentration [10]. The amount of methyl branches decreased with the increase of monomer concentration, while the amount of longer branches increased (Table 3). The increase of longer branches at high monomer concentration (1.5 mol/L) means that 1,2-insertion followed by successive monomer insertion is much faster than chain-walking (*ω*,2-enchainment, Scheme 1iii), which is well consistent with previous observation [10].

As shown in Table 3, it was found that the density of branches is primarily controlled by the length of the monomer employed [10]. Generally, the ratio of 1,2*- versus* 2,1-insertion is sensitive to the nature of the α-diimine ligand, this behavior was reported by Brookhart when using [NiBr_2_(IPPBIAN)]-MAO for the living polymerization of 1-hexene and 1-octene [4]. However, poly(1-alkene) produced by **C1**-MMAO exhibited a higher fraction of 1,2-insertions than polymers reported by Brookhart [4] and Guan [9], and poly(1-alkene)s exhibited lower fraction of *ω*,1-enchainment ranges from 22% to 42% (Table 3). The content of *ω*,1-enchainment decreased with increasing of monomer concentration, while the content of *ω*,1-enchainment increased with increasing of the polymerization temperature (entries 6–11, Table 3)

## 4. Conclusions

In summary, a series of α-diimine ligands bearing phenyl groups and their Ni(II) complexes were prepared and characterized. The ligands were modified in an attempt to change coordination environment, steric effects and the electronic density of the metal center, eventually to improve the activity in the polymerization of ethylene/1-alkene and to control the microstructure of polymers. Upon activation with either Et_2_AlCl or MMAO, these complexes showed high activities in ethylene polymerization and produced highly branched amorphous polyethylene (up to 145 branches/1000 C). Most interestingly, the *sec-*butyl branches were observed in polyethylene depending on reaction temperature and cocatalyst. Polymerization of 1-alkene (1-hexene, 1-octene, 1-decene and 1-hexadecene) with **C1**-MMAO at room temperature resulted in polyolefins with narrow *M*_w_/*M*_n_ values, which indicated a living polymerization. Living polymerization of 1-decene was observed at room temperature. These results indicate the possibility of precise microstructure control, depending on the polymerization temperature, monomer concentration and types of monomers, which in turn strongly affects the physical polymer properties.

## Figures and Tables

**Table 1:** Results of ethylene polymerization with nickel complexes **C1**–**C7**. *^a^*

Entry	cat.	co-cat.	[Al]/[Ni]	*T* (°C)	Yield (g)	act. ^b^	*M*_n_ *^c^* (×10^4^)	*M*_w_*/M*_n_ *^c^*	B ^d^
1	**C1**	Et_2_AlCl	200	20	1.10	1.32	13.4	1.68	–
2	**C1**	Et_2_AlCl	400	20	1.57	1.88	14.0	1.68	–
3	**C1**	Et_2_AlCl	600	20	1.73	2.08	16.3	1.73	105
4	**C1**	Et_2_AlCl	800	20	1.66	1.99	10.2	1.78	–
5	**C1**	Et_2_AlCl	1,000	20	1.45	1.74	12.9	1.82	–
6	**C1**	Et_2_AlCl	600	0	1.79	2.15	13.9	1.85	nd ^e^
7	**C1**	Et_2_AlCl	600	40	0.91	1.20	7.2	1.87	119
8	**C1**	Et_2_AlCl	600	60	0.56	0.67	3.3	1.87	134
9	**C1**	Et_2_AlCl	600	80	0.12	0.14	2.1	1.79	140
10	**C1**	MMAO	600	0	2.62	3.13	17.6	1.71	113
11	**C1**	MMAO	600	20	1.81	2.17	15.9	1.63	121
12	**C1**	MMAO	600	40	1.19	1.43	7.3	1.42	133
13	**C1**	MMAO	600	60	0.89	1.07	6.0	1.32	139
14	**C1**	MMAO	600	80	0.86	1.03	5.0	1.86	145
15	**C2**	MMAO	600	20	1.85	2.22	16.1	1.76	103
16	**C3**	MMAO	600	20	1.94	2.33	16.9	1.84	124
17	**C4**	MMAO	600	20	2.13	2.56	17.2	1.95	106
18	**C5**	MMAO	600	20	1.64	2.00	14.9	1.84	94
19	**C6**	MMAO	600	20	2.60	3.12	17.9	1.92	125
20	**C7**	MMAO	600	20	0.81	0.97	11.6	1.72	81

^a^ Polymerization conditions: Ni = 5.0 μmol; ethylene relative pressure 0.2 bar, ethylene absolute pressure 1.2 bar; time: 10 min; solvent = toluene (30 mL); ^b^ Activity was calculated as the 10^6^ g of polymer per mole of catalyst per hour, in unit of 10^6^ g PE/(mol Ni h); ^c^ *M*_n_ in 10^4^ g/mol, determined by GPC against polystyrene standard; ^d^ Branching density, branches/1000 C = (CH_3_/3)/[(CH + CH_2_ + CH_3_)/2] × 1000, determined by ^1^H NMR (Equation (S1)) [33]; ^e^ nd: not determined.

**Table 2:** Branched distributions of polyethylenes obtained by **C1**. ^a^

Entry/cocat.	*T* (°C)	*T*_g_ (°C)	Total CH_3_^b^	CH_3_/1000 C ^b^					
				Me	Et	Pr	Bu	*sec-*Bu	Lg (*n* ≥ 5)
3/Et_2_AlCl	20	–57.0	105	74	7	5	5	0	14
7/Et_2_AlCl	40	–58.8	120	84	8	3	6	1	18
10/MMAO	0	–56.7	114	88	7	1	3	1	14
11/MMAO	20	–58.3	122	88	9	3	3	2	17
12/MMAO	40	–60.6	131	86	11	2	6	7	19
13/MMAO	60	–65.5	143	82	15	3	8	12	23
14/MMAO	80	–68.2	146	88	16	2	8	10	22

^a^ Determined using DSC; ^b^ Branches per 1000 C, determined by ^13^C NMR (Equation (S3), Figures S12 and S16−S21).

**Table 3:** Polymerization of higher 1-alkene [1-hexene (H), 1-octene (O), 1-decene (D) and 1-hexadecene (HD)] with **C1**-MMAO . ^a^

Entry	Monomer		*T *(°C)	Yield (g)	act. ^b^	*M*_n_^c^ (×10^4^)	*M*_w_*/M*_n_^c^	B *^d^*	*ω*,1 ^e^ (%)	CH_3_/1000C ^f^				
	Type	[M]								Me	Pr	Bu	Lg	CH_3_
1	H	1.5	20	0.63	126	31.2	1.32	142	34	13	5	123	2	143
2	H	0.3	20	0.41	82	12.9	1.09	121	42	62	1	54	3	120
3	O	1.5	20	0.89	178	19.0	1.14	107	29	23	1		84	108
4	O	0.3	20	0.61	122	12.0	1.06	97	35	53	1		42	96
5	D	1.5	20	1.13	226	15.2	1.10	93	22	12			79	91
6	D	0.3	−20	0.12	24	4.2	1.18	91	23	2	0		89	86
7	D	0.3	0	0.23	46	5.2	1.08	87	26	18	1		67	85
8	D	0.3	20	0.68	136	10.8	1.09	85	27	40	2		39	81
9	D	0.3	40	0.56	112	12.3	1.22	81	30	44	2		33	79
10	D	0.3	60	0.41	82	6.7	1.33	79	32	46	4		26	76
11	D	0.3	80	0.23	43	3.7	1.41	76	34	51	4		18	73
12	HD	1.5	20	1.19	238	30.3	1.08	50	27	8			38	46
13	HD	0.3	0	0.37	74	6.4	1.06	48	30	3			40	43
14	HD	0.3	20	0.55	110	11.0	1.04	46	33	16			26	42

^a^ Polymerization conditions: Ni = 10 μmol; cocatalyst MMAO, [Al]/[Ni] = 300; higher 1-alkene = 10 mmol; 30 min; solvent: toluene; ^b^ Activity in kg polymer/(mol Ni h); ^c^ *M*_n_ in 10^4^ g/mol, determined by GPC against polystyrene standard; ^d^ Branches per 1000 C, which were determined by ^1^H NMR spectra; ^e^ *ω*,1-Enchainment was calculated by the following equation: *ω*,1% = [1000−(ω−2)B]/(1000 + 2B) (Equation (S2)); ^f^ From ^13^C NMR.

## Supplementary Materials

The following are available online at www.mdpi.com/2073-4360/8/4/160/s1. Molecular structures of the complexes **C1** and **C5** (Figures S1 and S2), crystal data of complex **C1** (Tables S1 and S2), NMR spectra of the organic compounds **1**–**3**, **L** and **L1**–**3** (Figures S3–S9), results of 1-decene polymerization (Table S3 and Figure S10), microstructure analysis of the polymers (Equations (S1)–(S3) and Table S4), NMR spectra of the polymers (Figures S12−S32), GPC and DSC curves of the polymers (Figures S11 and S33−S51).
