# Supplementary material for: Synthesis of Highly Branched Polyolefins Using Phenyl Substituted α-Diimine Ni(II) Catalysts
Source: Polymers (Basel). 2016 Apr 22;8(4):160. doi: 10.3390/polym8040160 (PMC6432043; doi:10.3390/polym8040160)
Supplement: Supplementary file 1 [file polymers-08-00160-s001.pdf]

Supplementary Materials: Synthesis of Highly Branched Polyolefins Using Phenyl Substituted α-Diimine Ni(II) Catalysts

Fuzhou Wang, Ryo Tanaka, Zhengguo Cai, Yuushou Nakayama and Takeshi Shiono

1. X-ray Structure Determinations

The molecular structures of complexes **C1** and **C5′** were confirmed by single-crystal X-ray diffraction and the corresponding ORTEP diagrams are shown in Figure S1. Crystal data, data collection, and refinement parameters are listed in Table S1. Selected bond distances and angles are summarized in Table S2.

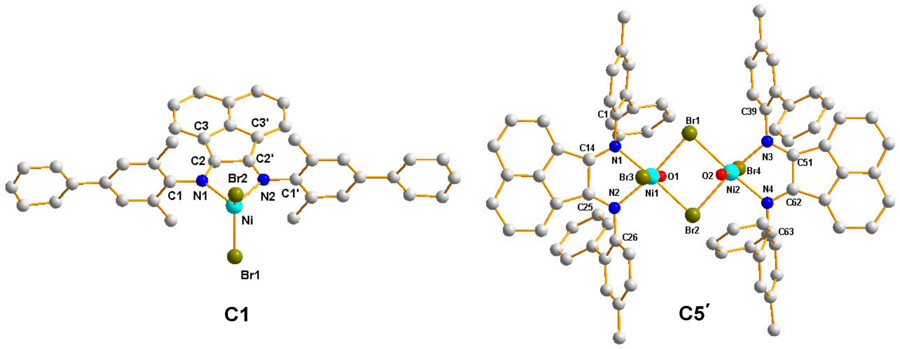

Figure S1. Molecular structures of the complexes **C1** and **C5′**, where H atoms are omitted for clarity.

Table S1. Crystal data and structure refinements of complex **C1**.

Identification Code	C1
Empirical formula	$C_{40}H_{32}Br_2N_2Ni$
Formula mass	759.21
Temperature (*K*)	292
Wavelength (Å)	0.7107
Crystal size (mm³)	0.33 × 0.31 × 0.28
Crystal system	Orthorhombic
Space group	*Cmcm*
a (Å)	11.7266 (8)
b (Å)	11.5635 (5)
c (Å)	27.5587 (14)
V (Å³)	3,737.0 (4)
Z	8
Density (calcd.) (mg/cm³)	2.699
Absorption coefficient (mm⁻¹)	5.37
F(000)	3,072
Theta range for data collec. (°)	3.0–28.5
Limiting indices	$-15 \leq h \leq 15$, $-14 \leq k \leq 15$, $-35 \leq l \leq 34$
Reflections collected	9,590
Independent reflections	1,900
R_{int}	0.056
Final *R* indices [$I > 2\sigma(I)$]	$R_1 = 0.0640$, $wR_2 = 0.1827$
R indices (all data)	$R_1 = 0.0945$, $wR_2 = 0.1986$
Refinement method	Full-matrix least-squares on F^2
Goodness-of-fit on F^2	1.137
Max. and min. transmission	1.0000 and 0.56405
Largest diff. peak and hole (e·Å⁻³)	0.482 and −0.301

Table S2. Selected bond lengths (Å) and angles (°) for complex **C1**.

Bond Lengths	(Å)	Bond Angles	(°)
Br1−Ni1	2.3343(12)	Br1−Ni1−Br1i	117.34(8)
Ni1−Br1i	2.3343(12)	N1ii−Ni1−Br1i	112.96(6)
Ni1−N1ii	2.041(5)	N1−Ni1−Br1i	112.96(6)
Ni1−N1	2.041(5)	N1−Ni1−Br1	112.96(6)
N1−C8	1.427(8)	N1ii−Ni1−Br1	112.96(6)
N1−C9	1.290(9)	N1ii−Ni1−N1	82.8(3)
C1−C2i	1.308(14)	C8−N1−Ni1	130.7(4)

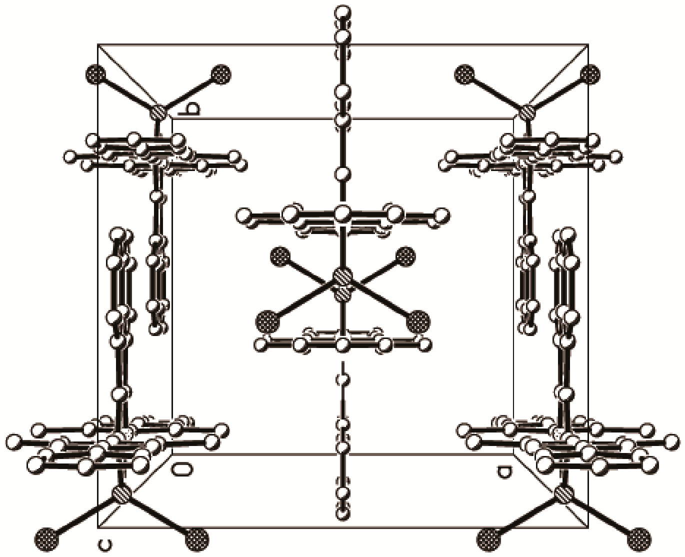

Figure S2. Cell structure of **C1** parallel to the bis(imino)acenaphthene unit.

2. Experimental Sections

*2.1. Synthesis and Characterizations of Complexes **C1**–**C7***

The synthesis of ligands **L**, **L1**–**L7** and their complexes **Pd-Cat.**, **C1**–**C7** are outlined in Scheme S1. After the protection of the amino group by acetic acid, the aniline derivatives were brominated. The Suzuki-coupling reaction of the bromoaniline derivatives and phenylboronic acid obtained with a Pd(II) catalyst (**Pd-Cat**.) in PEG-400/H_2O led to the corresponding phenyl-substituted aniline derivatives **1**–**6**. The ligands **L1**–**L7** were prepared by the condensation of two equivalents of the appropriate aniline with one equivalent of acenaphthoquinone, in the presence of a formic acid catalyst. The reaction of equimolar amounts of $NiBr_2$(DME) and the α-diimine ligands **L1**–**L7** in CH_2Cl_2 led to the displacement of 1,2-dimethoxyethane and afforded the Ni(II) complexes **C1**–**C7** as a moderately air-stable deep red microcrystalline solid in good yields, respectively. The corresponding palladium dichloride complex (**Pd-Cat.**) is accessible by the reaction of the *ortho*-*sec*-phenethyl substituted chiral ligand **L** with $(CH_3CN)_2PdCl_2$ in CH_2Cl_2 at ambient temperature. Compounds **L1**–**L7** were well characterized by IR, ^{1}H NMR, ^{13}C NMR spectroscopy and elemental analysis.

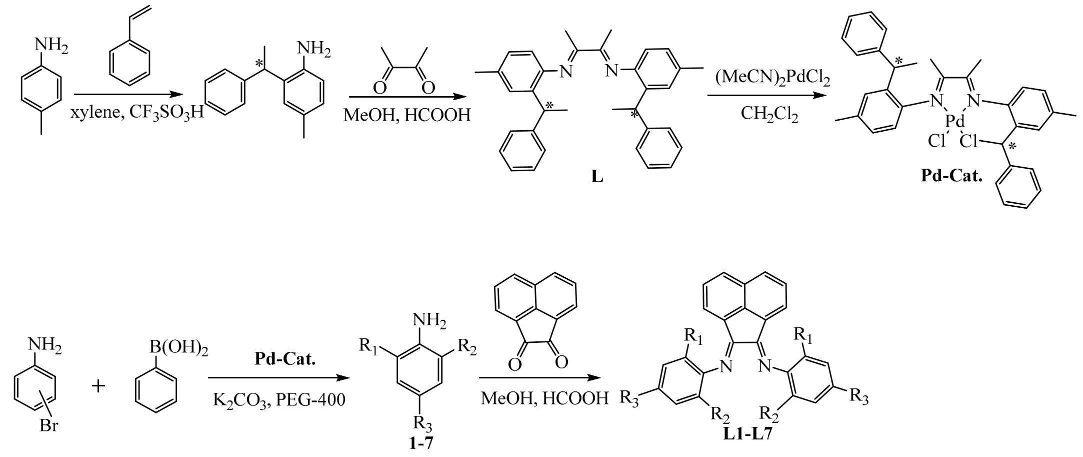

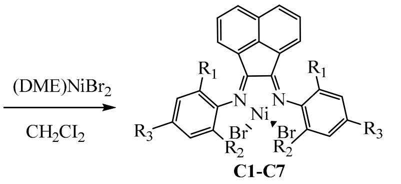

	L1 C1	L2 C2	L3 C3	L4 C4	L5 C5	L6 C6	L7 C7
R_1	Me	Me	Me	Ph	H	Ph	Me
R_2	Me	Ph	Ph	Ph	Ph	Ph	Me
R_3	Ph	Me	Ph	Me	Me	Ph	Me

Scheme S1. Synthesis of α-diimine ligands **L**, **L1–L7** and their complexes **Pd-Cat.**, **C1–C7**.

The new organic compounds were well characterized by ^{1}H NMR and ^{13}C NMR (see below).

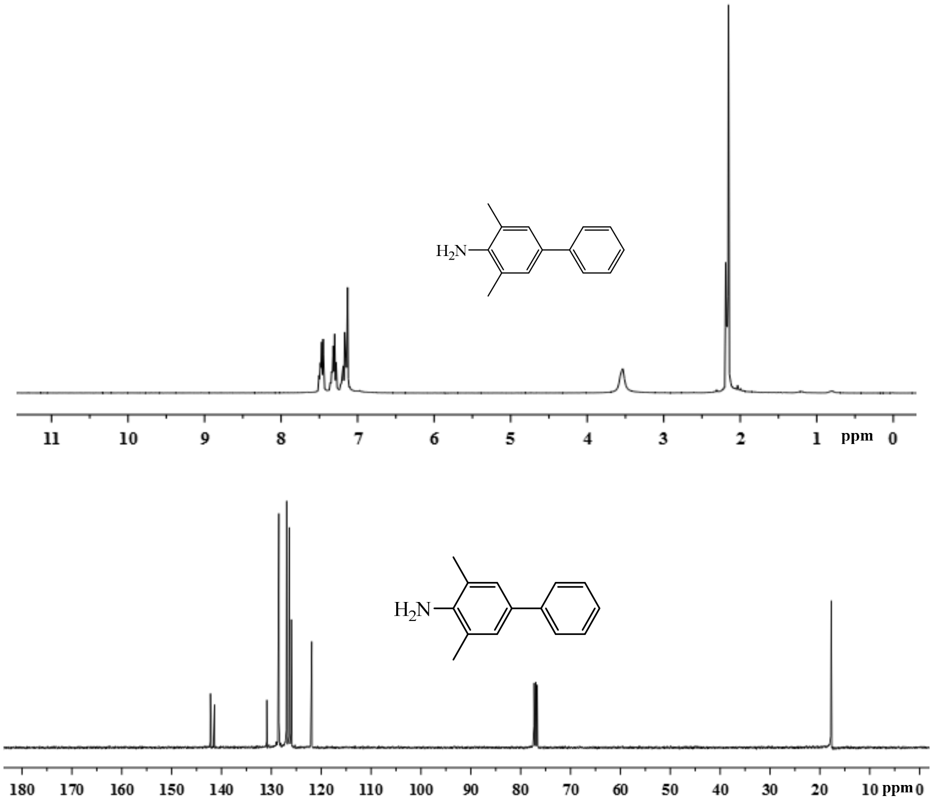

Figure S3. ^{1}H and ^{13}C NMR spectra of **1**.

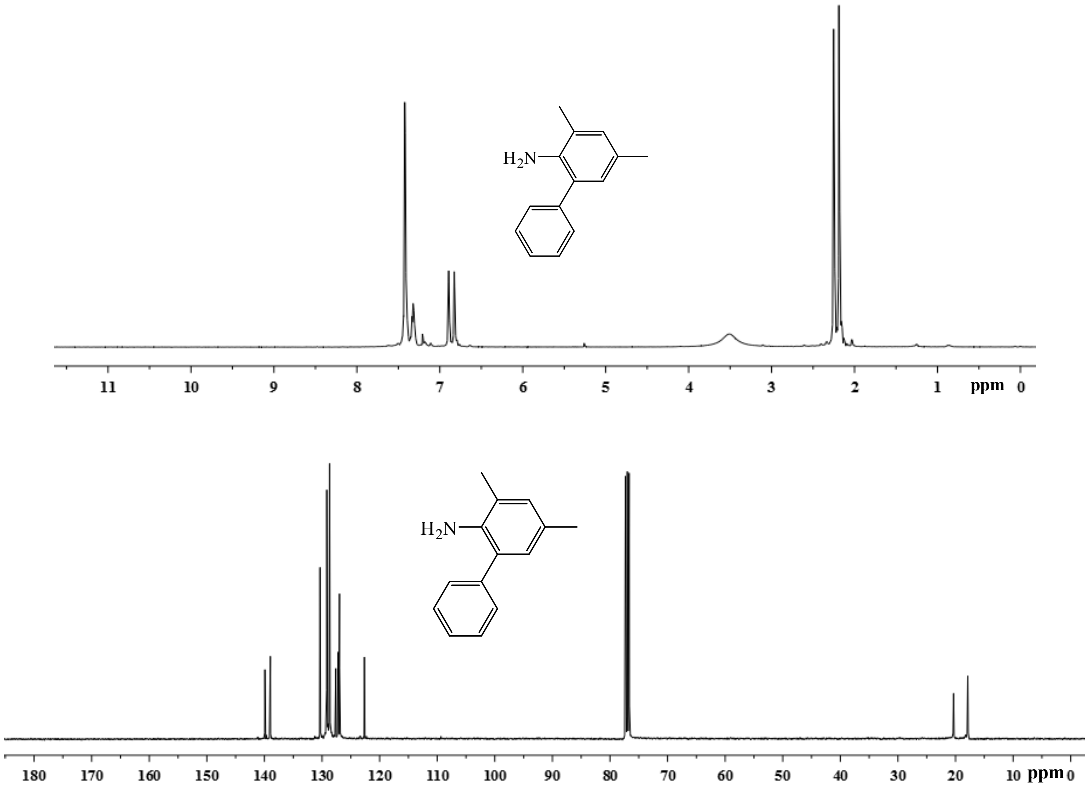

Figure S4. ^{1}H and ^{13}C NMR spectra of **2**.

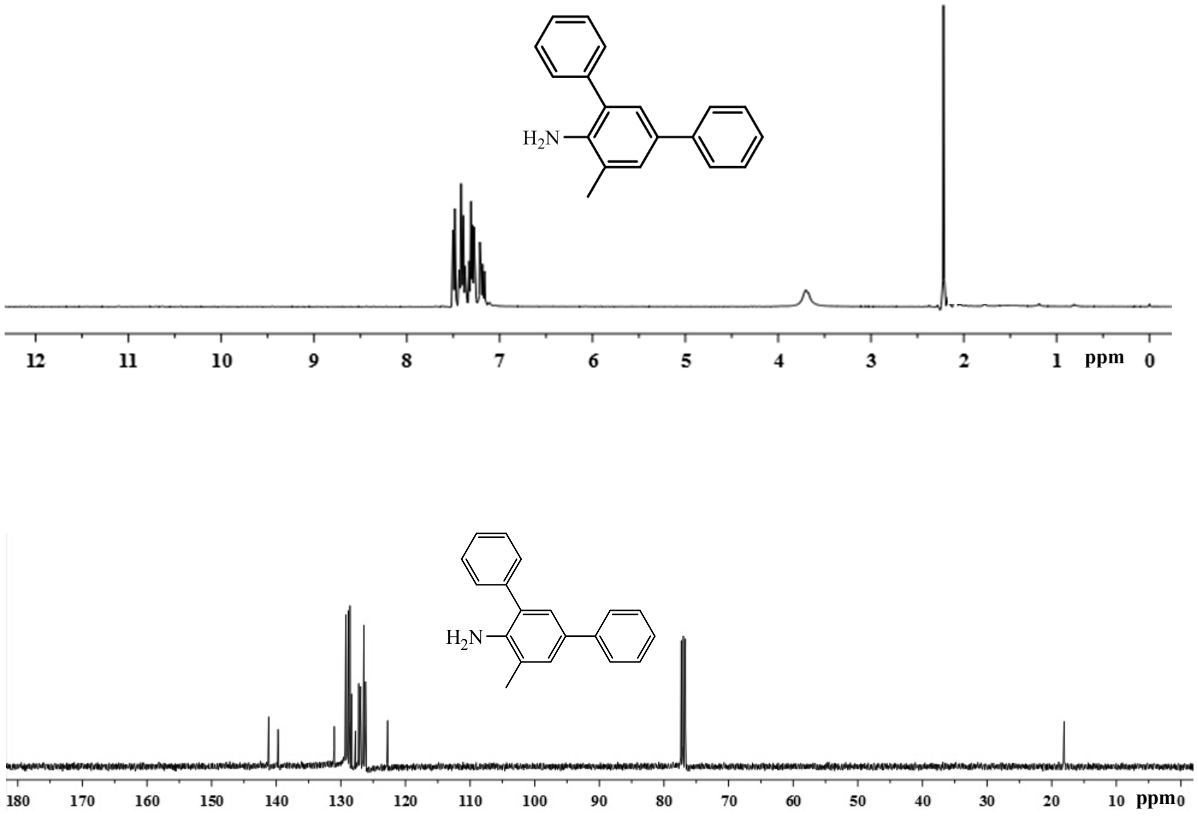

Figure S5. ^{1}H and ^{13}C NMR spectra of **3**.

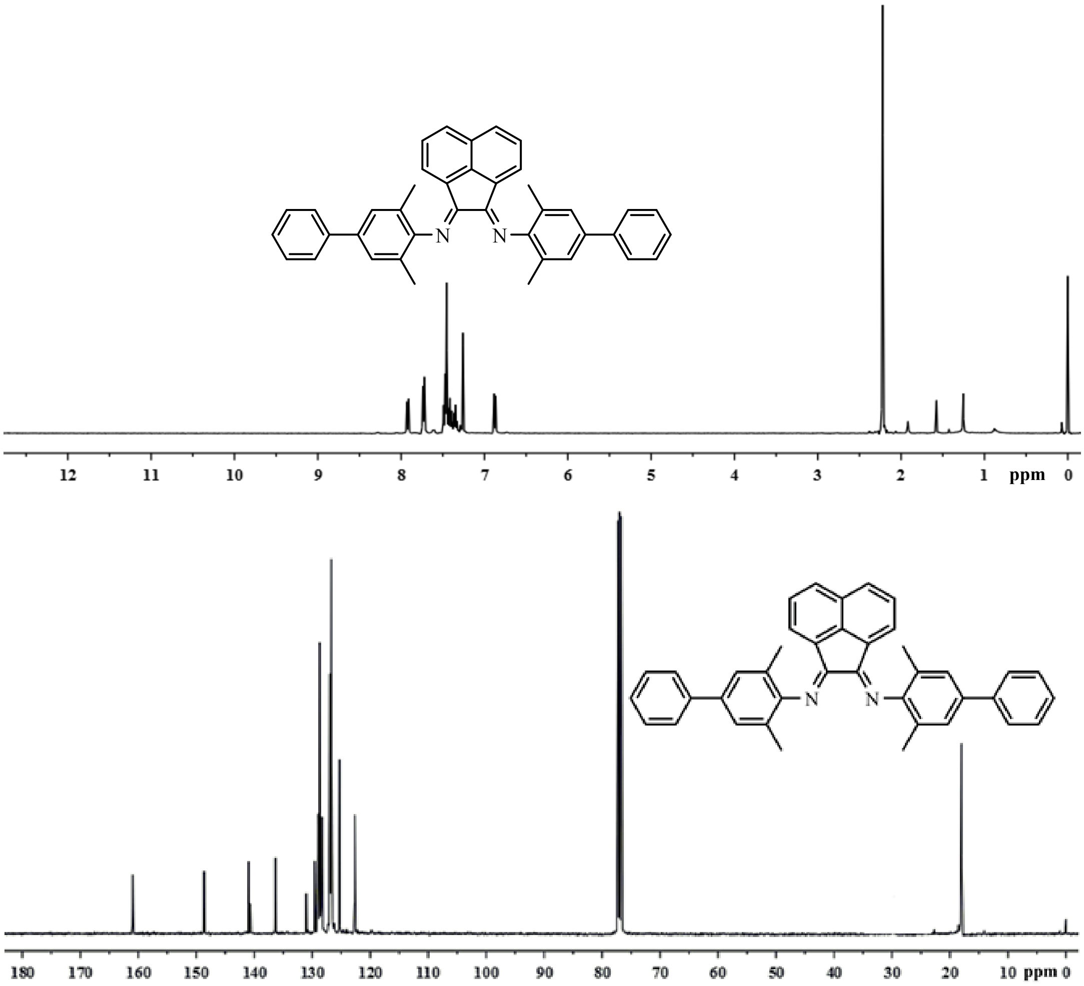

Figure S6. ^{1}H and ^{13}C NMR spectra of ligand **L1**.

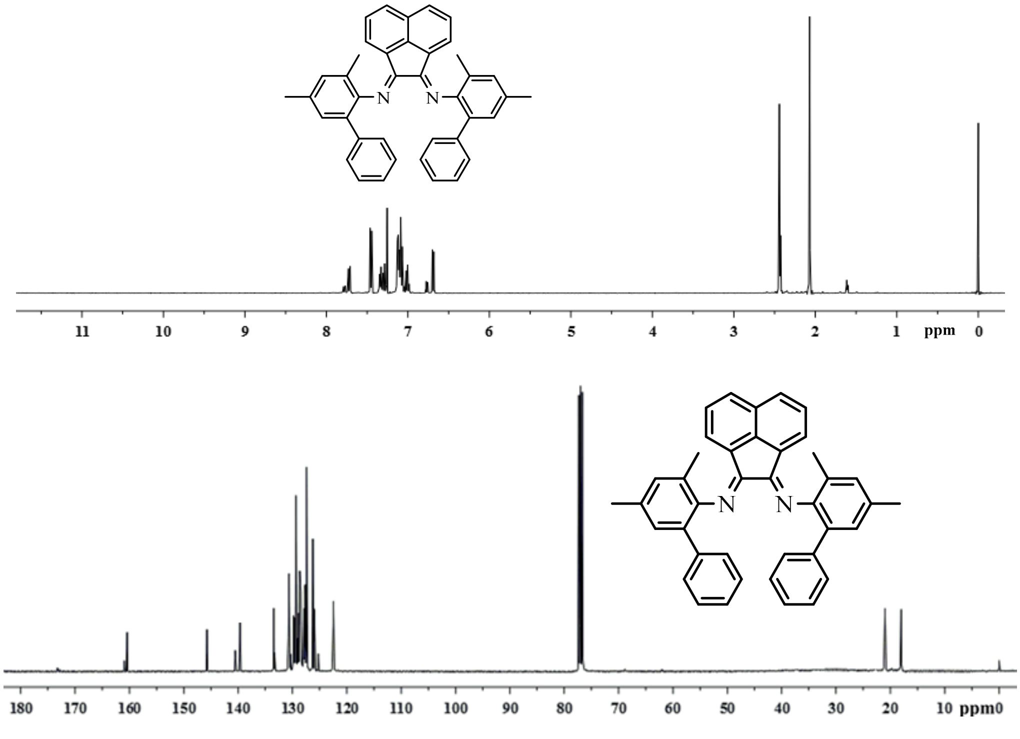

Figure S7. ^{1}H and ^{13}C NMR spectra of ligand **L2**.

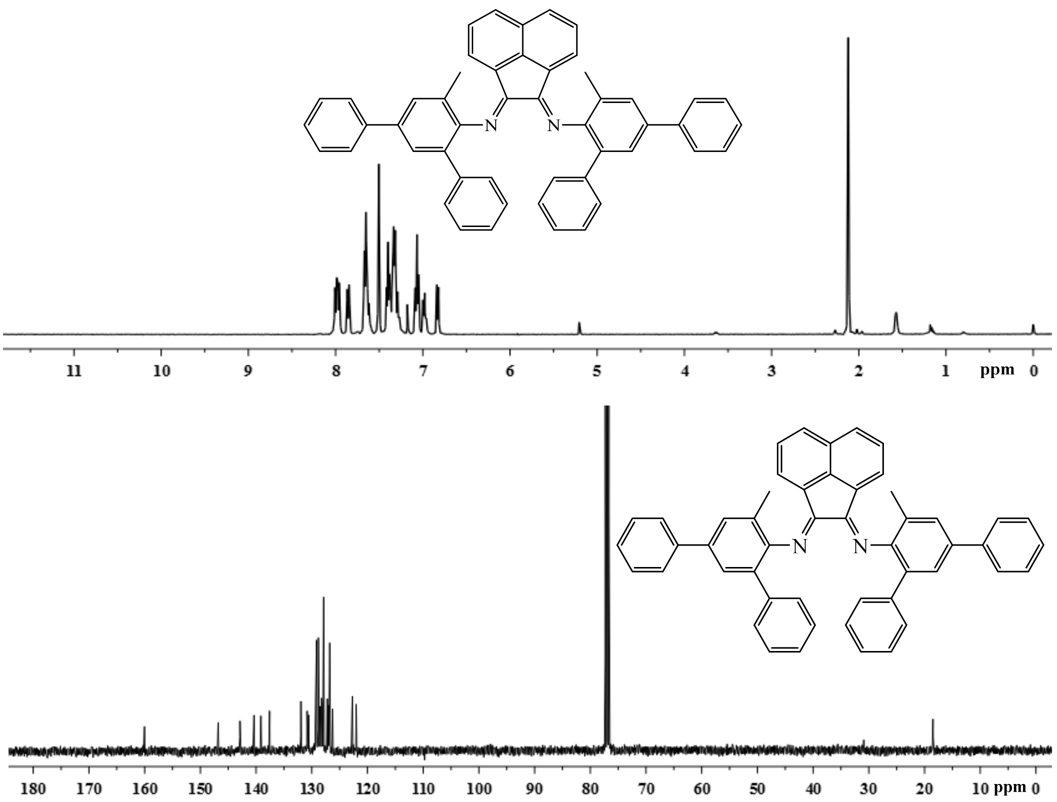

Figure S8. ^{1}H and ^{13}C NMR spectra of ligand **L3**.

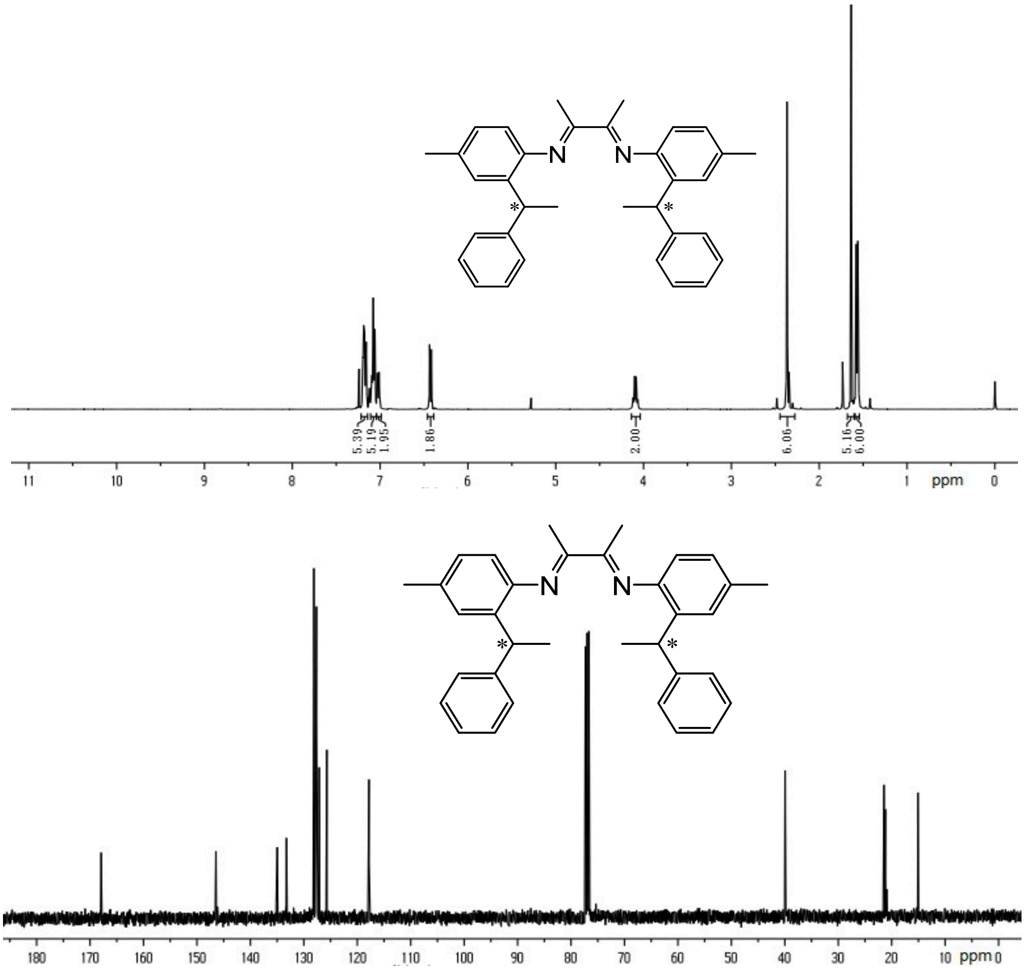

Figure S9. ^{1}H and ^{13}C NMR spectra of ligand **L**.

2.2. Living Polymerization of 1-Decene with C1-MMAO

Polymerization of 1-decene was investigated with **C1**-MMAO at 20 °C with the [Al]/[Ni] molar ratio of 300, and the results are summarized in Table S3.

Table S3. Polymerization of 1-decene with **C1**-MMAO. [a]

Entry	Time (min)	Conversion (%)	M_n [b] (kg·mol^{-1})	M_w/M_n [b]	N [c] (mol)
1	5	7.3	18.8	1.09	5.4
2	15	19.5	51.4	1.08	5.3
3	30	45.4	110.0	1.07	5.8
4	40	58.1	151.8	1.10	5.4
5	50	75.1	186.9	1.15	5.6
6	60	91.3	233.5	1.14	5.5

[a] Polymerization conditions: Ni = 10 µmol; cocatalyst MMAO, [Al]/[Ni] = 300; 1-decene = 10 mmol; 20 °C; solvent = toluene, total volume = 20 mL; [b] M_n in kg/mol, determined by GPC against polystyrene standard. [c] Number of polymer chain calculated by yield and M_n.

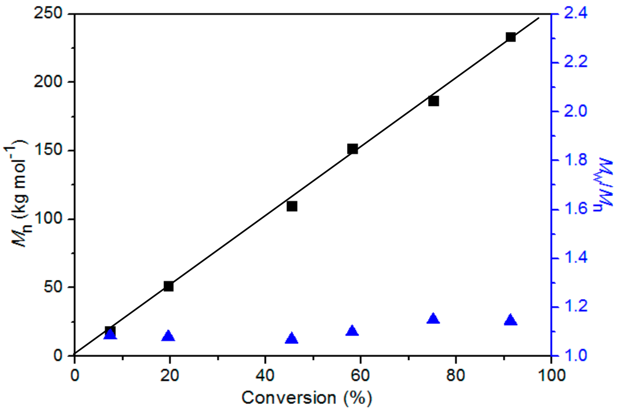

Figure S10. Plots of M_n (■) and M_w/M_n (▲) as a function of conversion for the polymerization of 1-decene (20 °C, Table S3).

GPC traces for the poly(1-decene)s (Table S3):

5 min

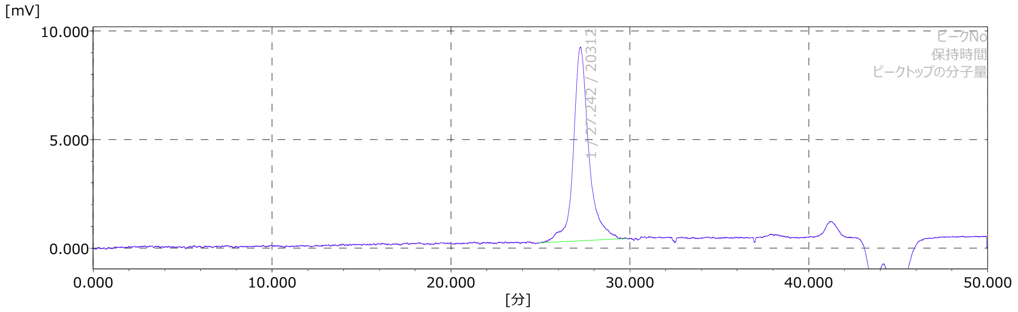

Poly(1-decene) (5 min, 20 °C, entry1, Table S3)

: クロマト RI (-0.955 - 10.185 [mV])

- RI - Mn: 18784 Mw: 20390 Mz: 22224 Mw/Mn: 1.086

15 min

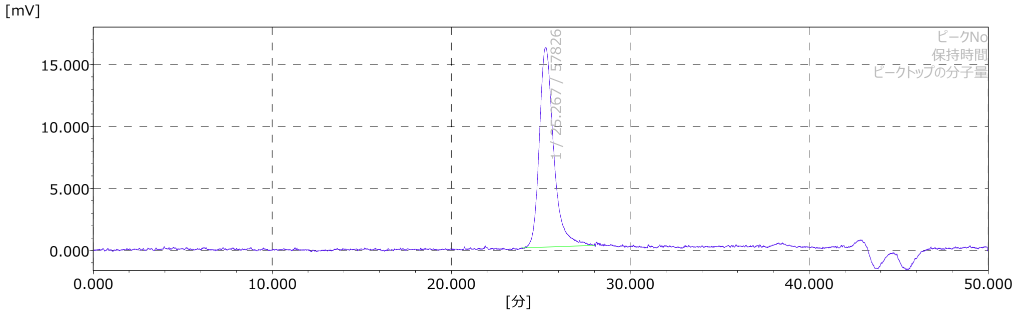

Poly(1-decene) (15 min, 20 °C, entry 2, Table S3)

: クロマト RI (-1.614 - 17.995 [mV])

- RI - Mn: 51370 Mw: 55278 Mz: 58505 Mw/Mn: 1.076

30 min

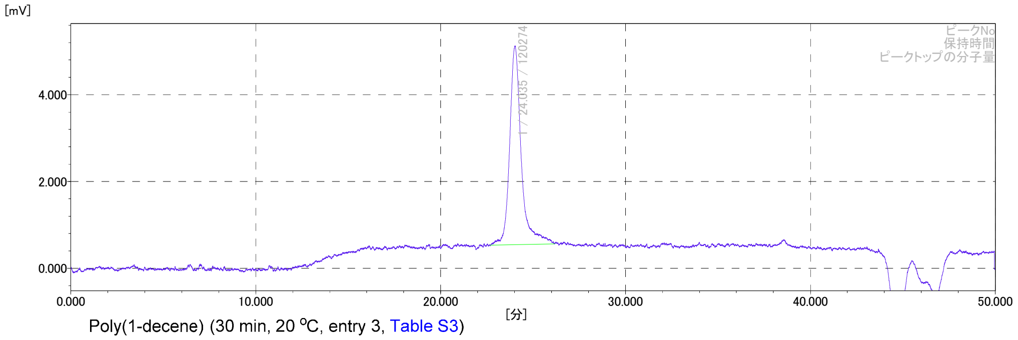

Poly(1-decene) (30 min, 20 °C, entry 3, Table S3)

: クロマト RI (-0.517 - 5.637 [mV])

- RI - Mn: 110042 Mw: 117614 Mz: 123862 Mw/Mn: 1.069

40 min

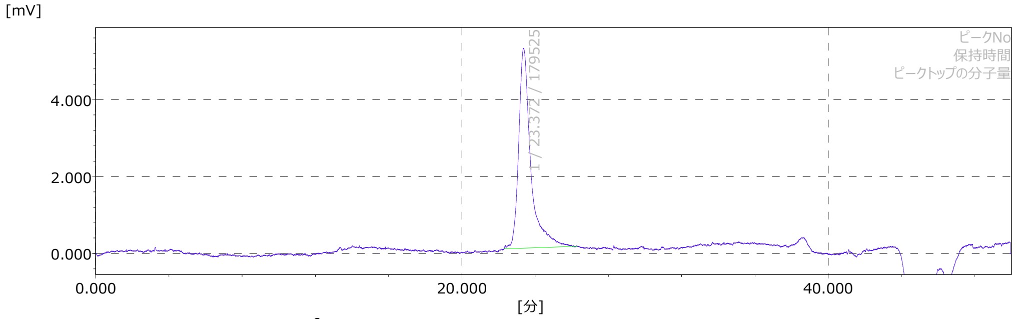

Poly(1-decene) (40 min, 20 °C, entry 4, Table S3)

: クロマト RI (-0.548 - 5.873 [mV])

- RI - Mn: 151809 Mw: 166903 Mz: 177948 Mw/Mn: 1.099

Figure 11. *Cont.*

50 min

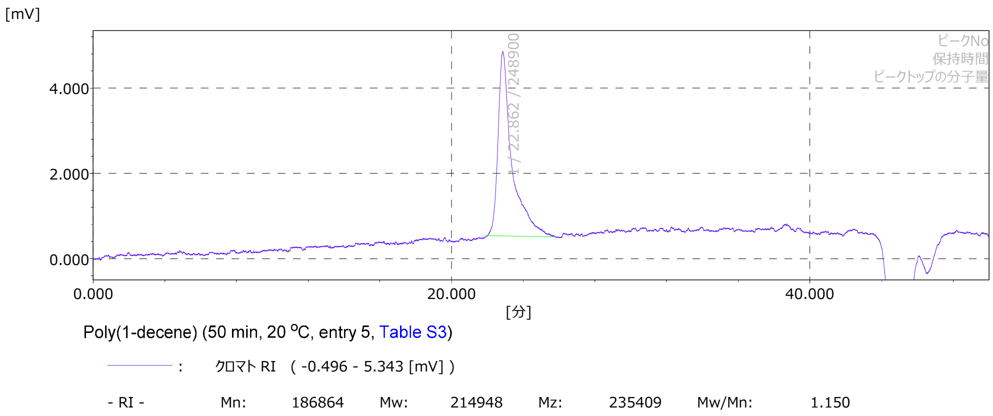

60 min

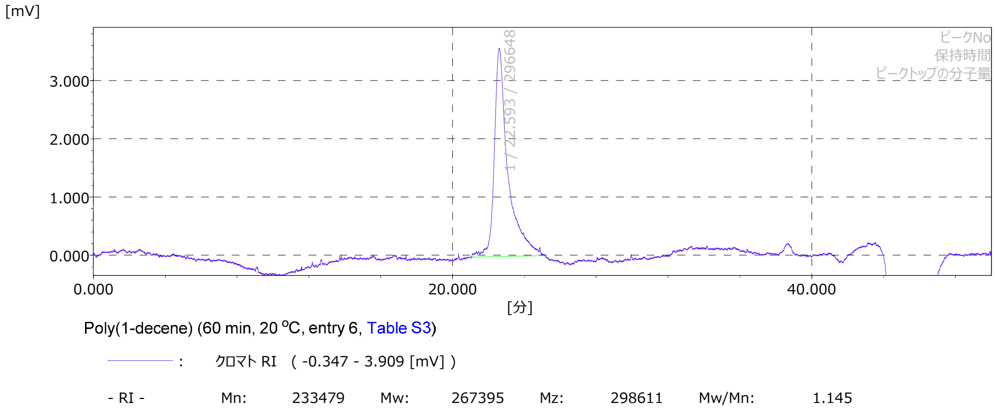

Figure S11. GPC traces for the poly(1-decene)s obtained with **C1**-MMAO at different reaction time (entries 1–5, Table S3).

3. Microstructure Analysis of the Polymers

3.1. Equations (S1)–(S3) for the Microstructure Analysis of the Polymers

3.1.1. Calculation of the Degree of Branching

The degree of branching (B) was estimated by ^{1}H NMR spectroscopy and was corrected for end groups as follows (S1):

$$B = \frac{2(I_{CH_3})}{3(I_{CH} + I_{CH_2} + I_{CH_3})} \times 1000 \quad \text{(S1)}$$

Branching degree, the number of methyl carbon in every 1000 carbons, CH_3, CH_2, CH refer to the intensities of the methyl, methylene and methine resonances in ^{1}H NMR spectra.

3.1.2. Calculation of ω,1-Insertions

The fraction of *ω*,1-insertions was calculated using the following equation reported by Brookhart *et al.* [1].

$$\omega,1\% = \frac{1000 - (\omega - 2)B}{1000 + 2B} \times 100 \quad \text{(S2)}$$

where B is the total branching calculated by Equation (S1), ω is the number of carbon atoms in the monomer.

Assignments of the ^{13}CNMR spectra and equations for the quantitative analysis of the polyolefins under investigation, according to Equation (S3) reported by Azoulay *et al.* [2]. Chemical shift and assignment of the peak listed in the Table S4 are marked in the spectrum (Figure S12).

$$\mathbf{CH_3} = (I_2 + I_5)/2 + I_1 + I_3 + I_6$$

$$\text{Methyl branch, } \mathbf{Me} = I_6 - I_4 + I_7\text{; Ethyl branch, } \mathbf{Et} = (I_1 + I_{10})/2 \quad \text{(S3)}$$

$$\text{Propyl branch, } \mathbf{Pr} = (I_4 + I_{23})/2\text{; Butyl branch, } \mathbf{Bu} = I_3 - I_8$$

$$sec\text{-Butyl branch, } {}^{s}\mathbf{Bu} = (I_2 + I_5)/2\text{; Longer branch, } \mathbf{Lg} = (I_8 + I_{17})/2$$

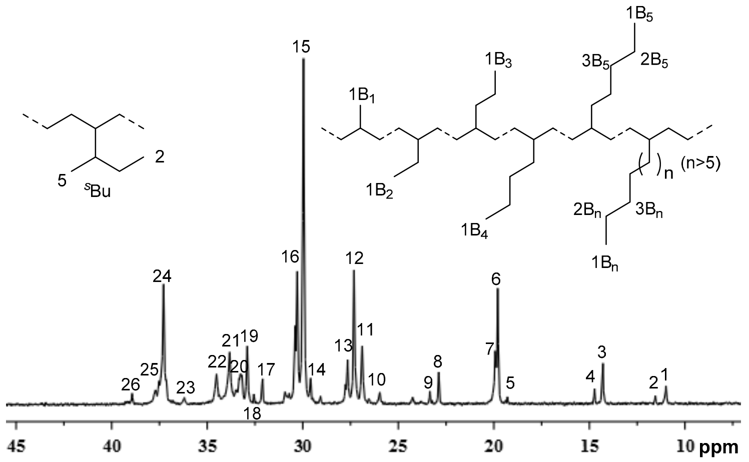

Figure S12. ^{13}C NMR spectrum of the polyethylene obtained with **C1**-MMAO at 20 °C (entry 11, Table 1).

Table S4. Chemical shift and assignment of peaks. [a]

Peak No.	Chemical shift (ppm)	Assignment	Peak No.	Chemical shift (ppm)	Assignment
1	11.09	$1B_2$	14	29.61	$4B_n$
2	11.53	^{s}Bu	15	29.95	CH_2
3	14.25	$1B_4$, $1B_n$	16	30.36	γB_1, γB_{2+}
4	14.66	$1B_3$	17	32.09	$3B_n$
5	19.33	^{s}Bu	18	32.57	$3B_5$
6	19.81	$1B_1$	19	32.94	brB_1
7	19.98	$2B_3$	20	33.34	αB_2
8	22.85	$2B_n$	21	33.91	αB_{3+}
9	23.24	$2B_4$	22	34.56	nB_n
10	26.03	$2B_2$	23	36.84	$3B_3$
11	26.95	$(n\text{-}1)B_n$	24	37.33	αB_1
12	27.30	βB_{2+}	25	37.77	brB_n
13	27.66	βB_1	26	39.03	brB_2

[a] ^{13}C NMR ($CDCl_3$ δ: 77.16 ppm) measurements of the polyethylene are marked in Figure S12; Note on labels: for xB_n B_n is a branch of length n carbons, *x* is the carbon being discussed, and the methyl at the end of the branch is numbered 1. Thus, the second carbon from the end of a butyl branch is $2B_4$. xB_{n+} refers to branches of length n and longer.

3.2. ^1H and ^{13}C NMR Spectra of the Polyethylenes (Tables 1 and 2)

NMR spectra of the polyethylenes prepared by **C1**–**C7** (see Figures S13–S21):

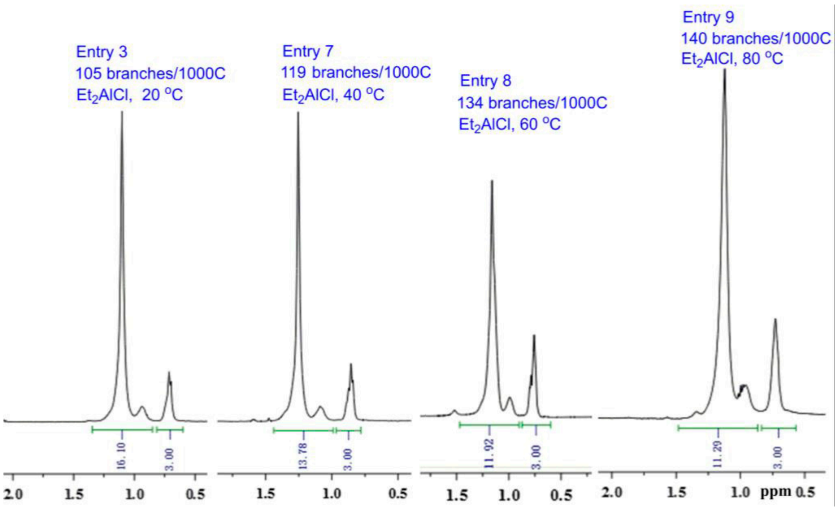

Figure S13. ^1H NMR spectra of the polyethylenes obtained with **C1**-Et_2AlCl (entries 3 and 7–9, Table 1).

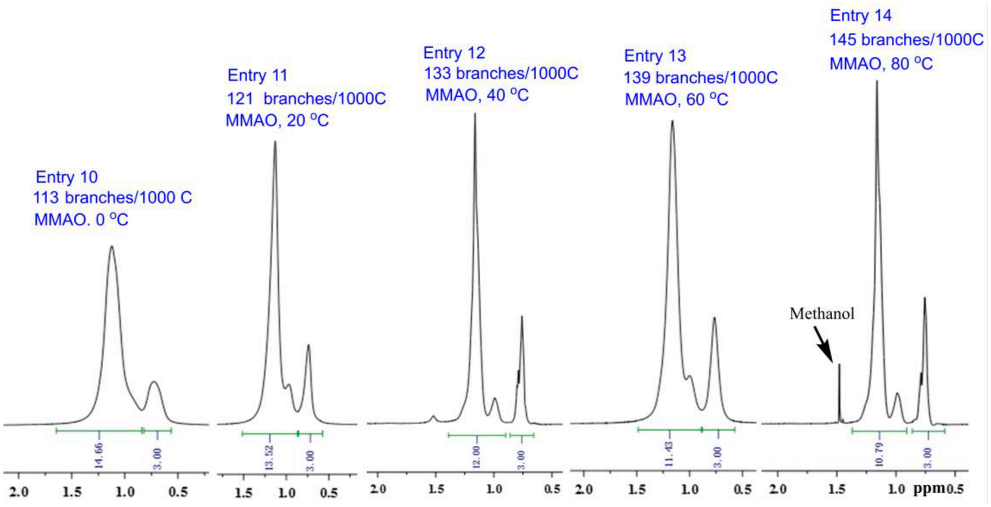

Figure S14. ^1H NMR spectra of the polyethylenes obtained with **C1**-MMAO (entries 10–14, Table 1).

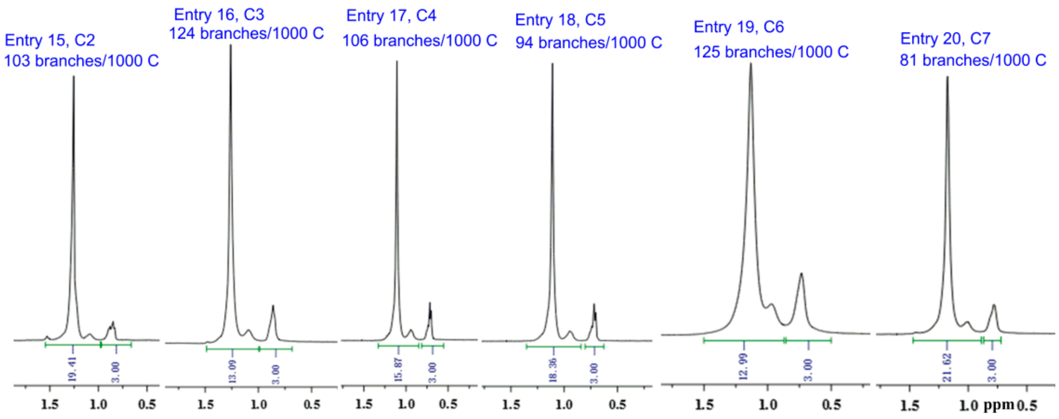

Figure S15. ^1H NMR spectra of the polyethylenes obtained with **C2**–**C7**/MMAO (entries 15–20, Table 1).

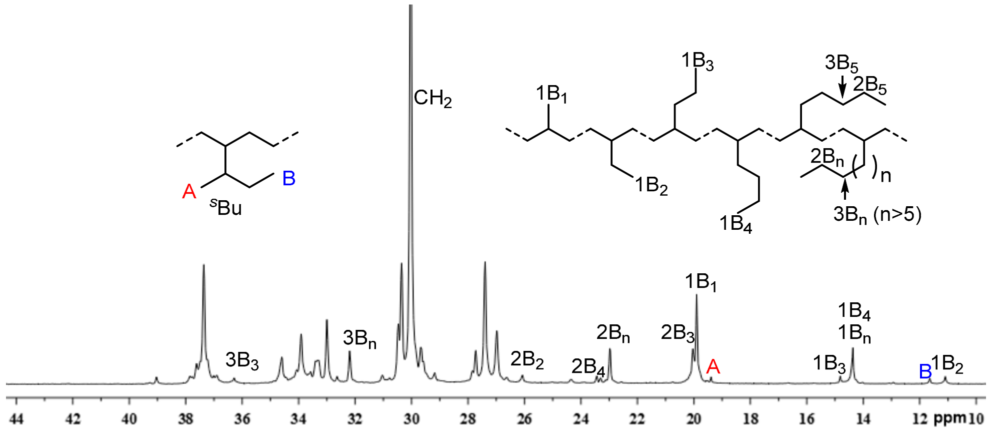

Figure S16. ^{13}C NMR spectra of the polyethylene obtained with **C1**-Et2AlCl at 40 °C (entry 7, Table 1).

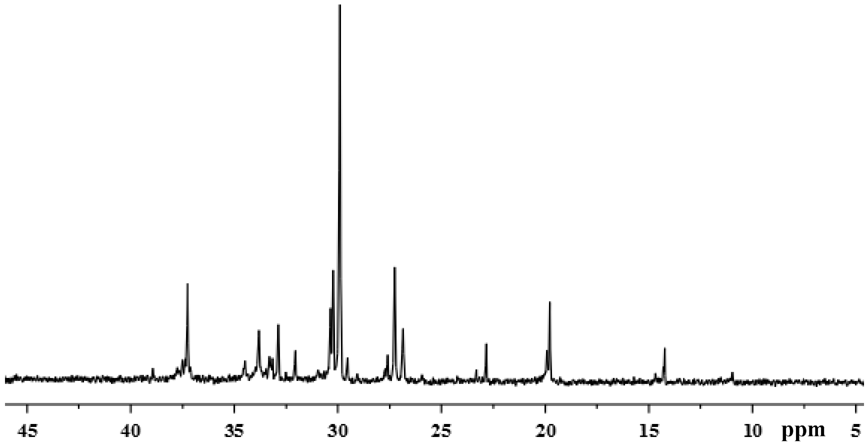

Figure S17. ^{13}C NMR spectra of the polyethylene obtained with **C1**-Et2AlCl at 20 °C (entry 3, Table 1).

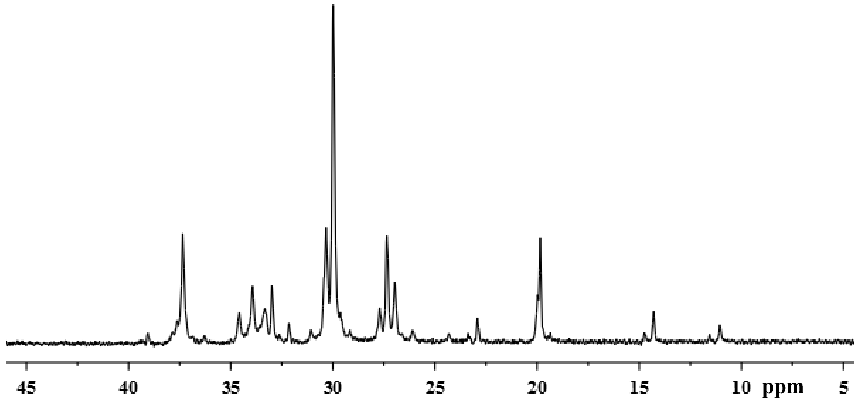

Figure S18. ^{13}C NMR spectra of the polyethylene obtained with **C1**-MMAO at 0 °C (entry 10, Table 1).

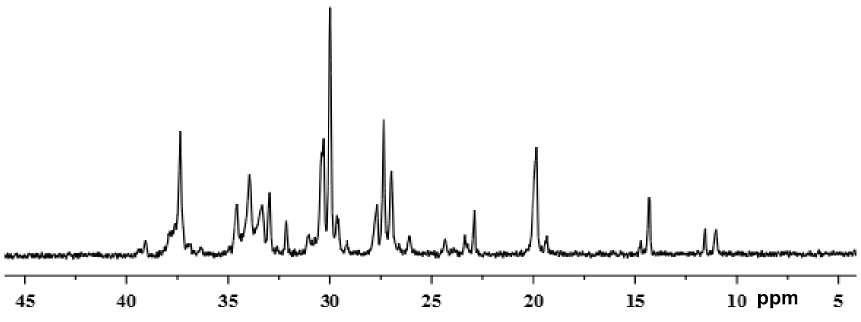

Figure S19. ^{13}C NMR spectra of the polyethylene obtained with **C1**-MMAO at 40 °C (entry 12, Table 1).

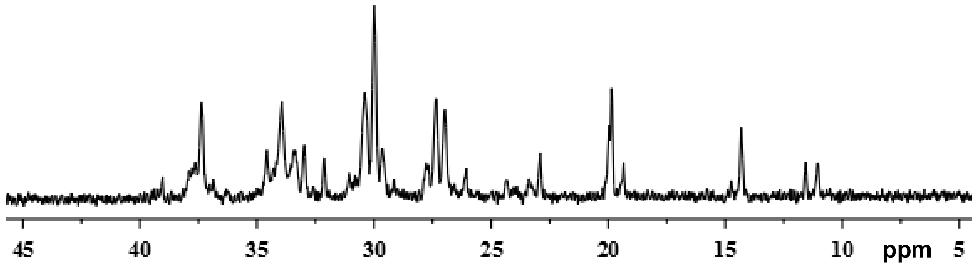

Figure S20. ^{13}C NMR spectra of the polyethylene obtained with **C1**-MMAO at 60 °C (entry 13, Table 1).

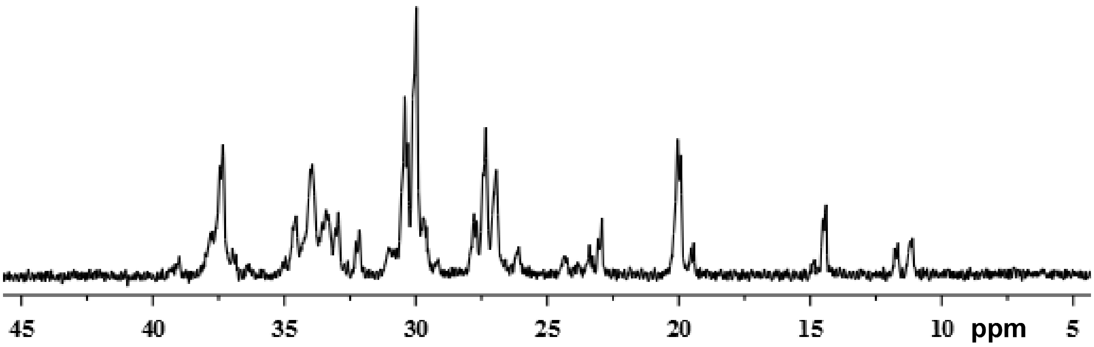

Figure S21. ^{13}C NMR spectra of the polyethylene obtained with **C1**-MMAO at 80 °C (entry 14, Table 1).

3.3. ^{1}H and ^{13}C NMR Spectra of the Poly(1-Alkene)s (Table 3)

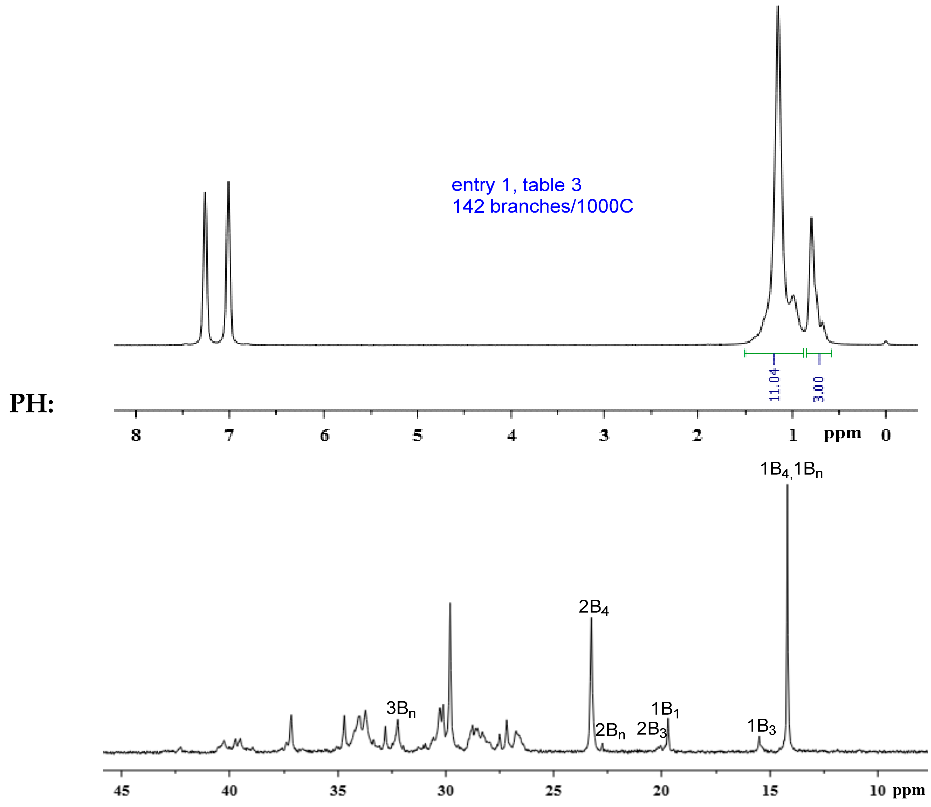

Figure S22. ^{1}H and ^{13}C NMR spectra of poly(1-hexene) obtained with **C1**-MMAO at 20 °C (entry 1, Table 4).

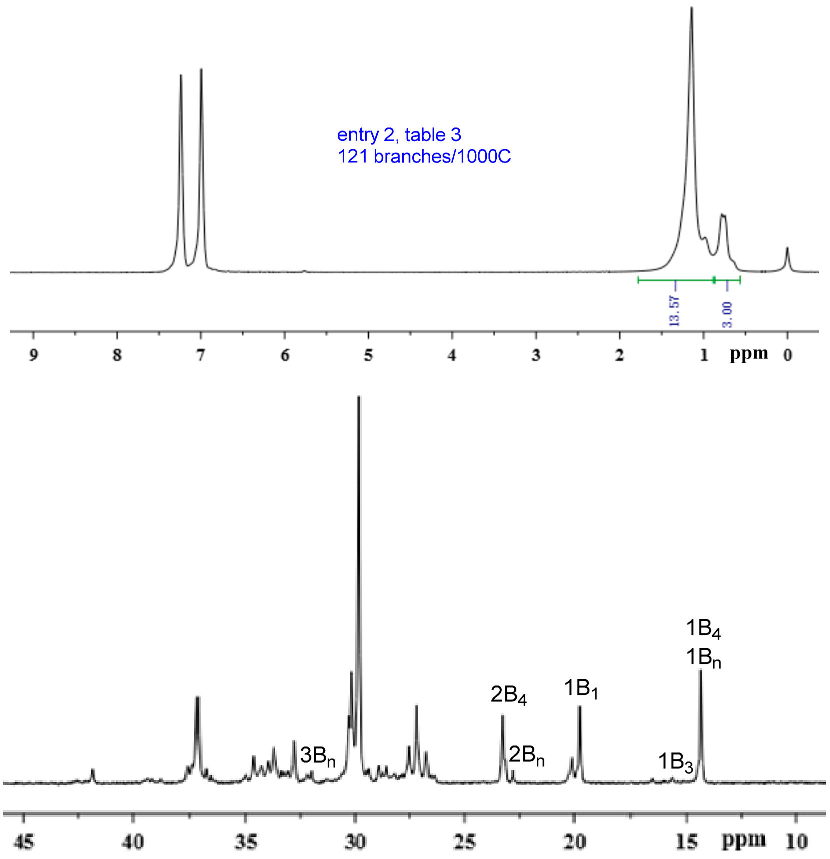

Figure S23. ^{1}H and ^{13}C NMR spectra of poly(1-hexene) obtained with **C1**-MMAO at 20 °C (entry 2, Table 3).

PO:

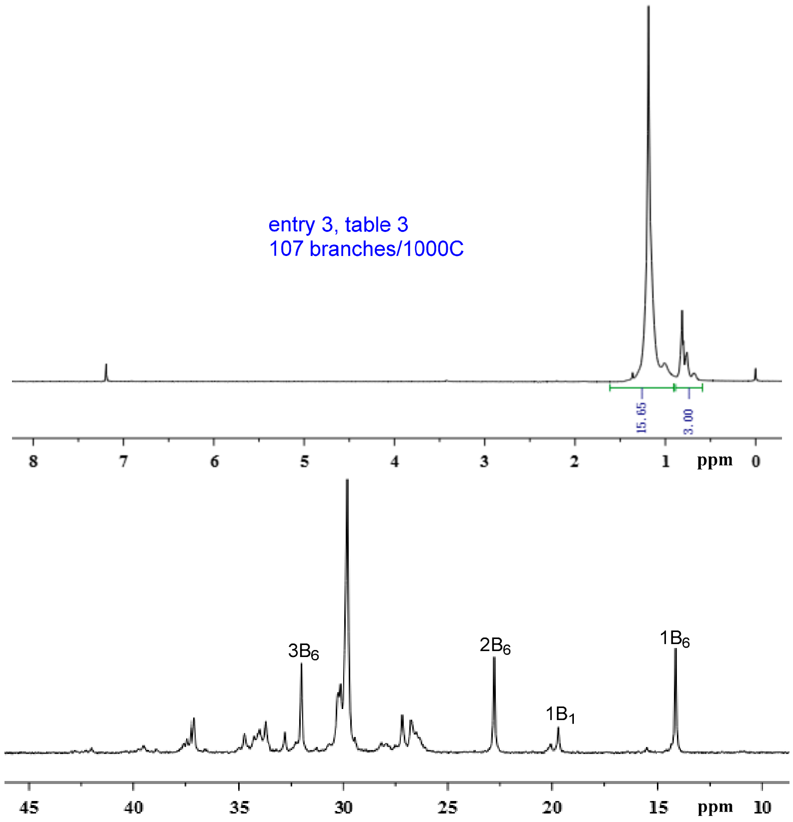

Figure S24. ^{1}H and ^{13}C NMR spectra of poly(1-octene) obtained with **C1**-MMAO at 20 °C (entry 3, Table 3).

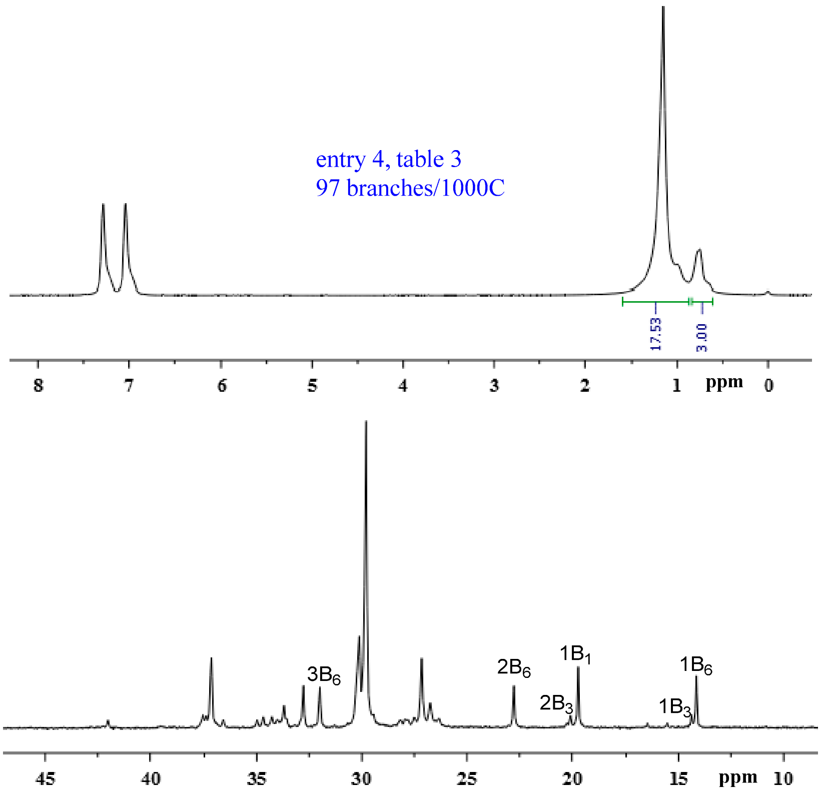

Figure S25. ^{1}H and ^{13}C NMR spectra of poly(1-octene) obtained with **C1**-MMAO at 20 °C (entry 4, Table 3).

PD:

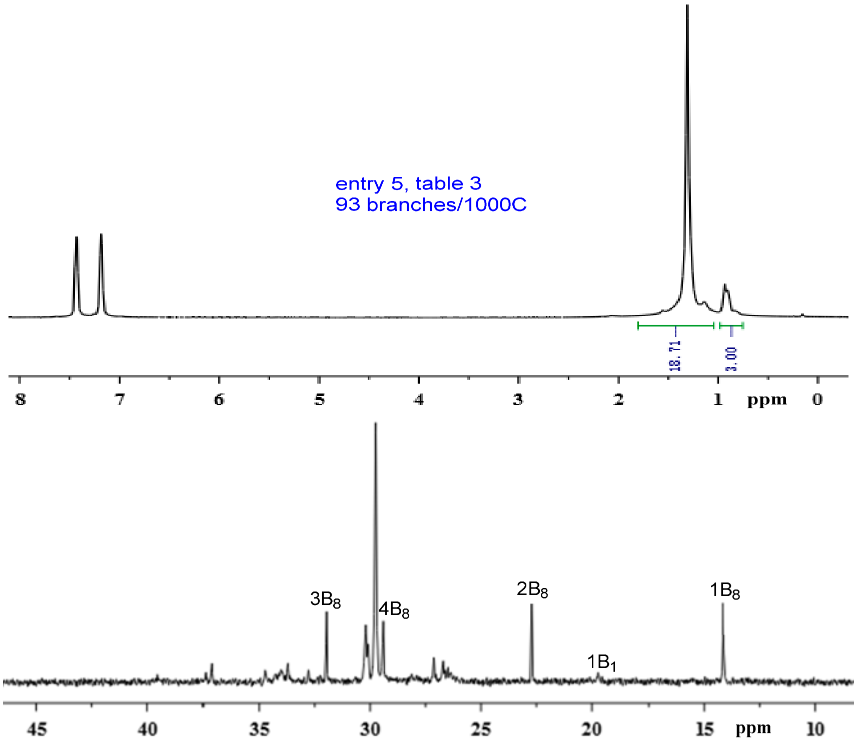

Figure S26. ^{1}H and ^{13}C NMR spectra of poly(1-decene) obtained with **C1**-MMAO at 20 °C (entry 5, Table 3).

PD:

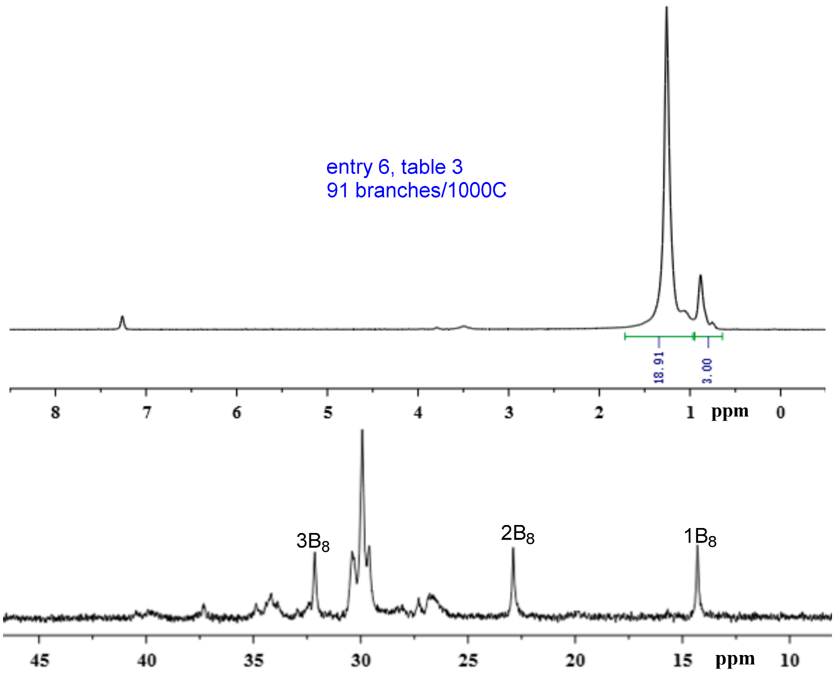

Figure S27. ^{1}H and ^{13}C NMR spectra of poly(1-decene) obtained with **C1**-MMAO at −20 °C (entry 6, Table 3).

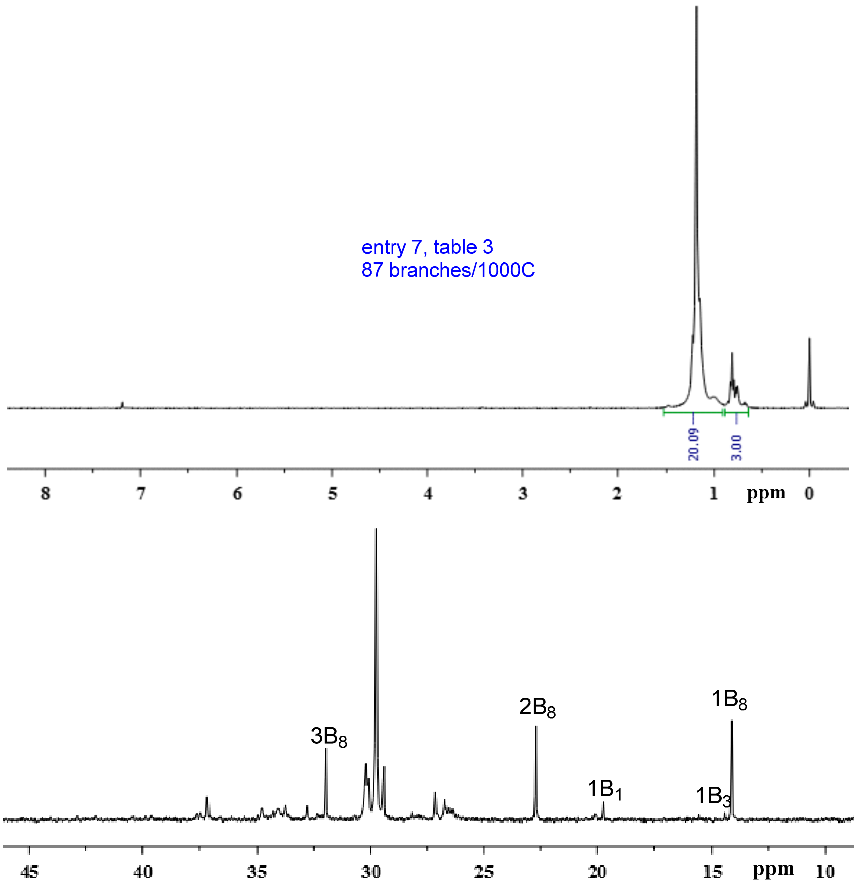

Figure S28. ^{1}H and ^{13}C NMR spectra of poly(1-decene) obtained with **C1**-MMAO at 0 °C (entry 7, Table 3).

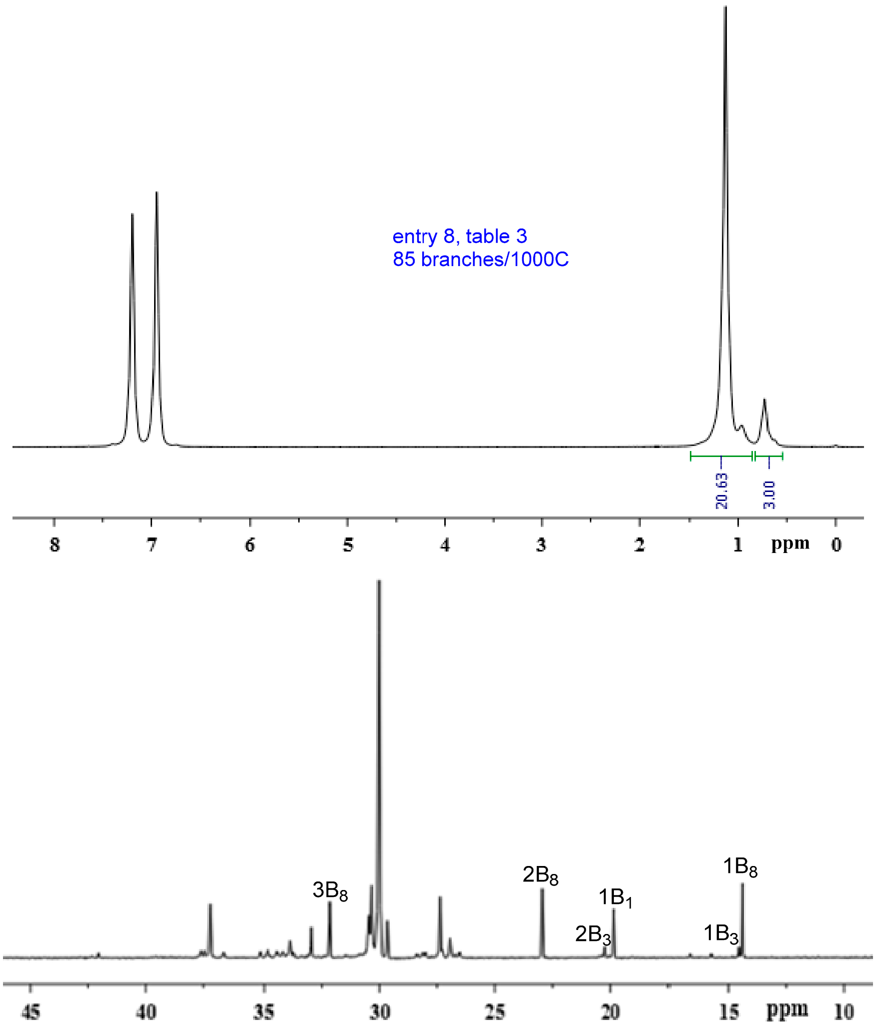

Figure S29. ^1H and ^{13}C NMR spectra of poly(1-decene) obtained with **C1**-MMAO at 20 °C (entry 8, Table 3).

PHD:

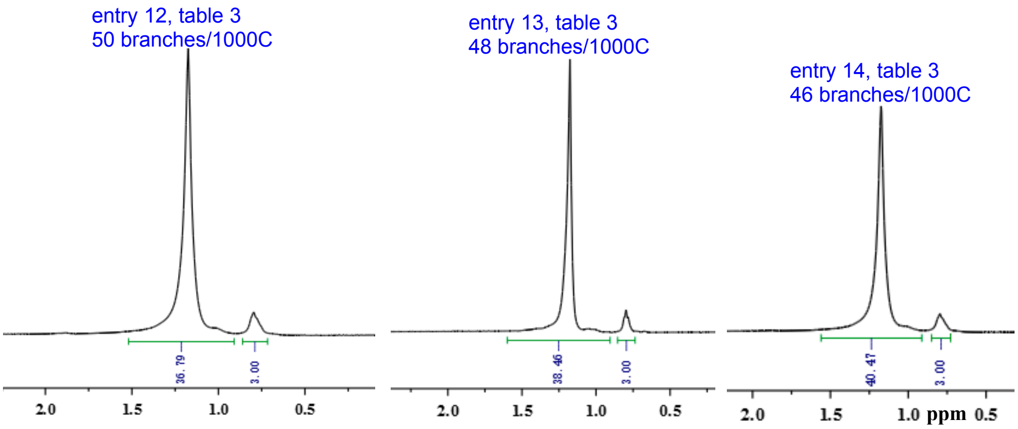

Figure S30. ^1H NMR spectra of poly(1-hexadecene)s obtained with **C1**-MMAO at 20 °C (entries 12–14, Table 3).

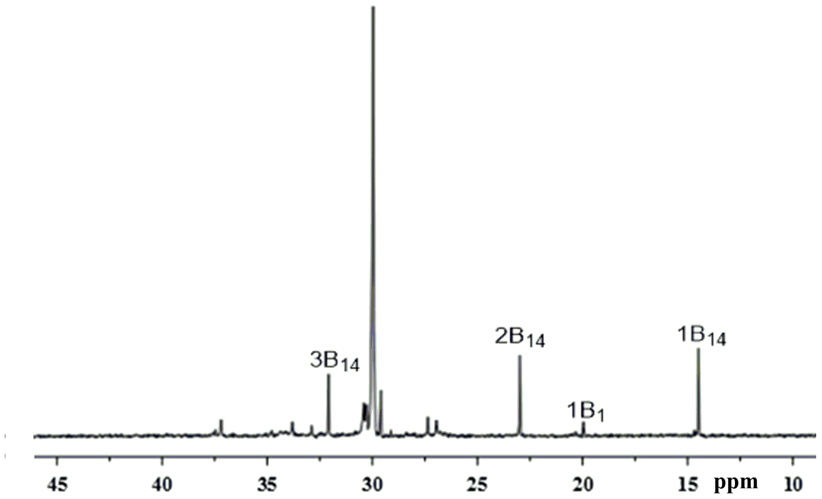

Figure S31. ^{13}C NMR spectra of poly(1-hexadecene) obtained with **C1**-MMAO at 0 °C (entry 13, Table 3).

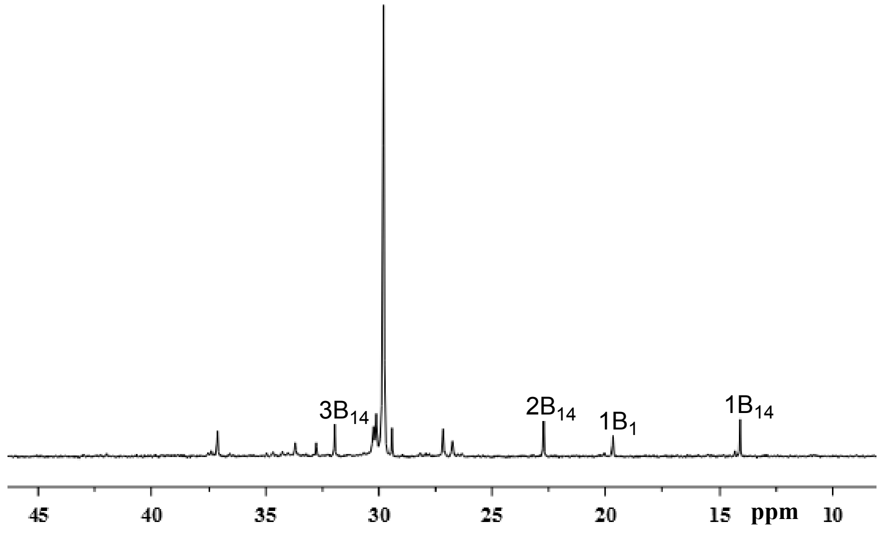

Figure S32. ^{13}C NMR spectra of poly(1-hexadecene) obtained with **C1**-MMAO at 20 °C (entry 14, Table 3).

4. GPC Traces of the Polymers

PE:

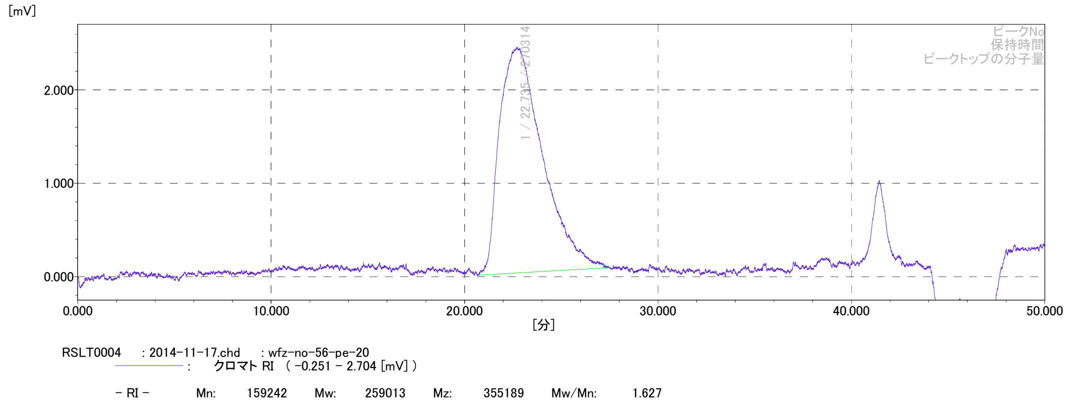

Figure S33. GPC trace for the polyethylene obtained with **C1**-MMAO at 20 °C (entry 11, Table 1).

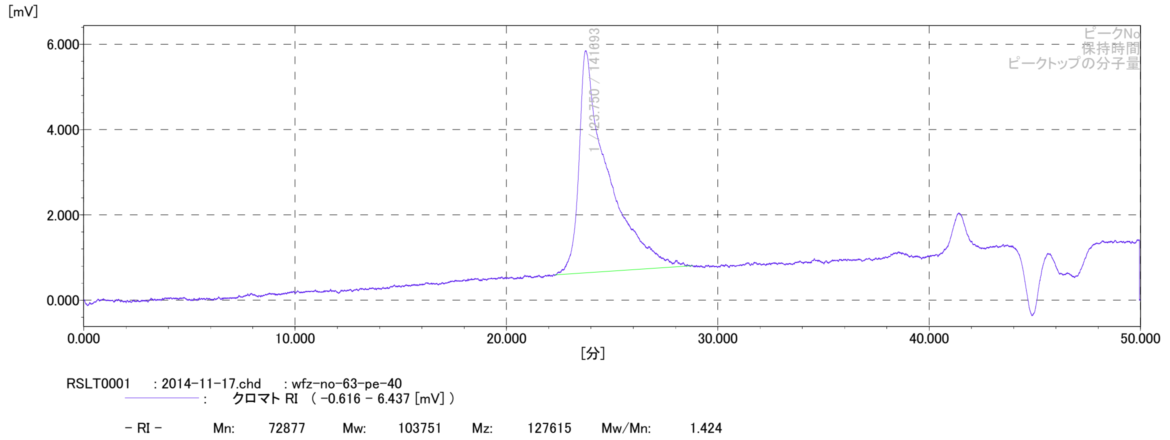

Figure S34. GPC trace for the polyethylene obtained with **C1**-MMAO at 40 °C (entry 12, Table 1).

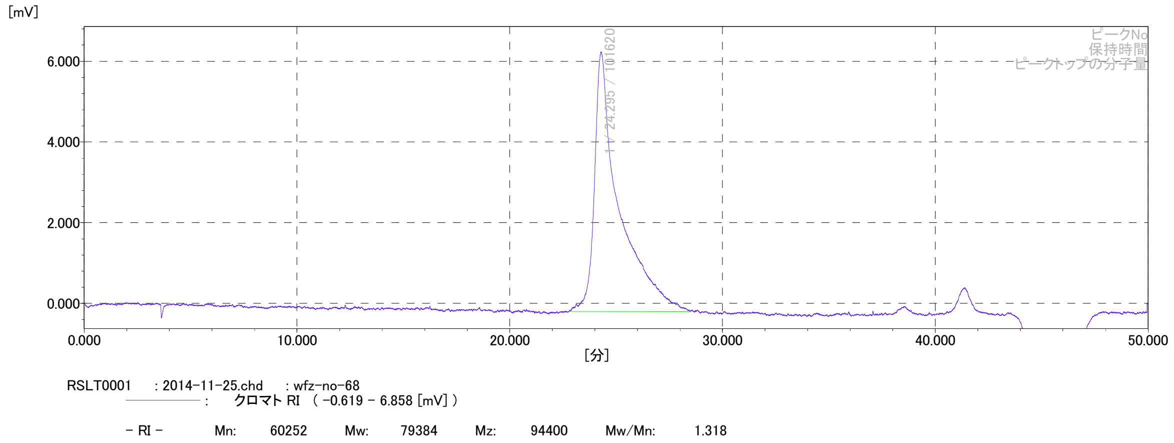

Figure S35. GPC trace for the polyethylene obtained with **C1**-MMAO at 60 °C (entry 13, Table 1).

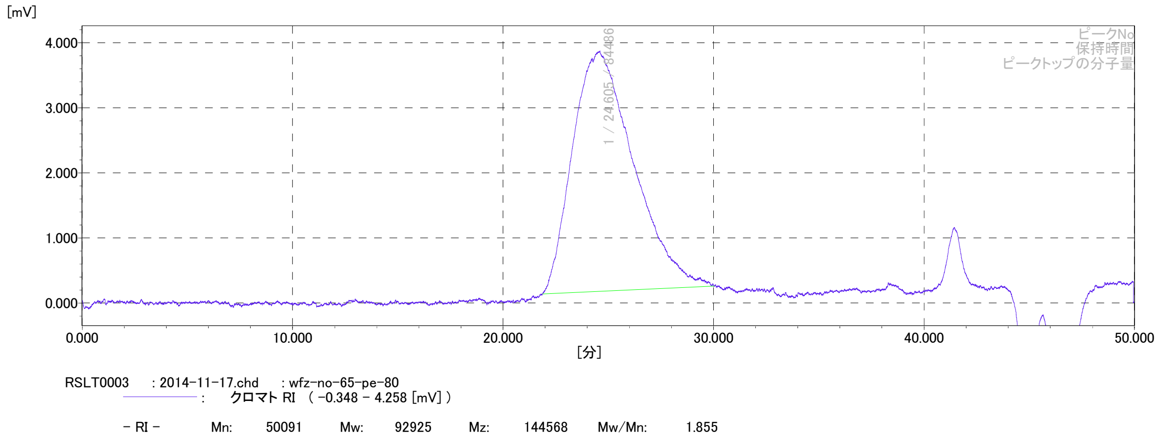

Figure S36. GPC trace for the polyethylene obtained with **C1**-MMAO at 80 °C (entry 14, Table 1).

PH:

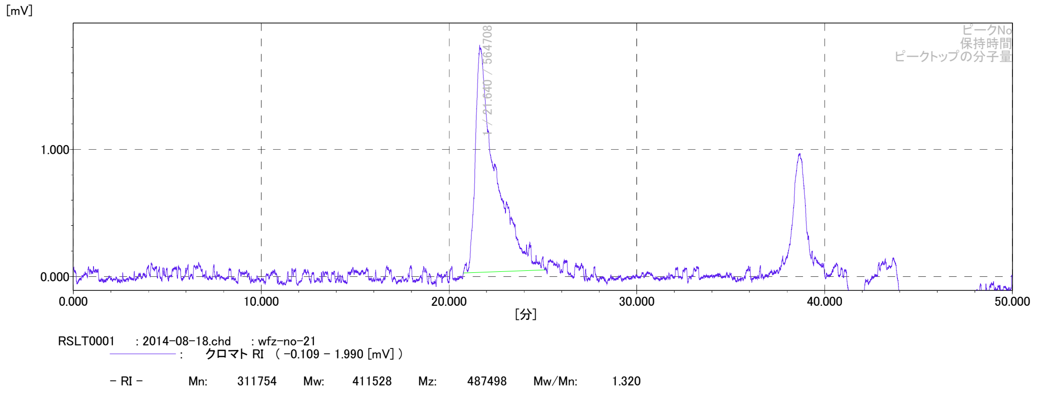

Figure S37. GPC trace for the poly(1-hexene) obtained with **C1**-MMAO at 20 °C for 30 min (entry 1, Table 3).

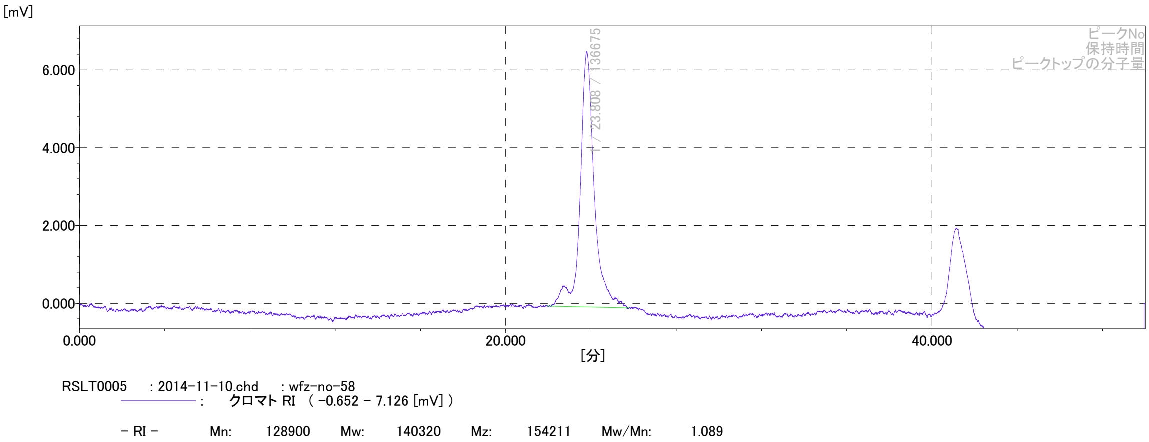

Figure S38. GPC trace for the poly(1-hexene) obtained with **C1**-MMAO at 0 °C for 30 min (entry 2, Table 3).

PO:

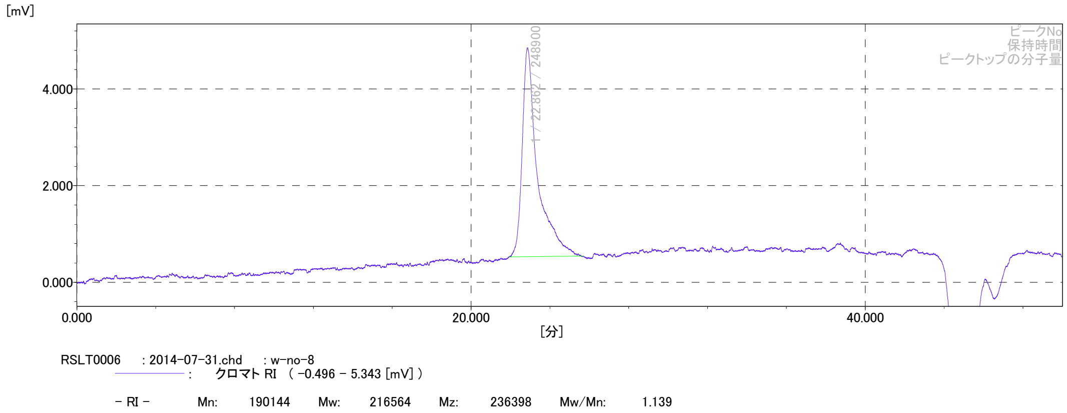

Figure S39. GPC trace for the poly(1-octene) obtained with **C1**-MMAO at 20 °C for 30 min (entry 3, Table 3).

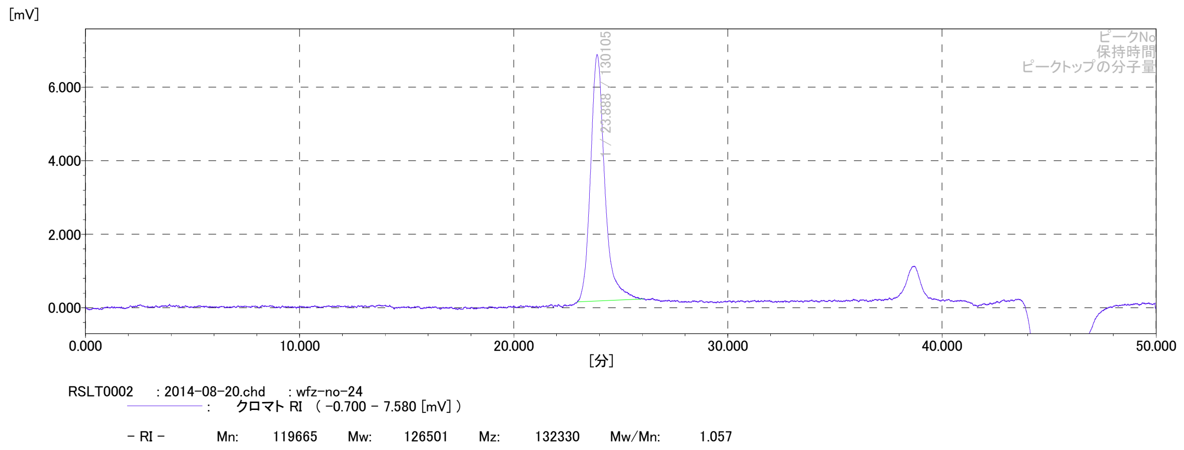

Figure S40. GPC trace for the poly(1-octene) obtained with **C1**-MMAO at 0 °C for 30 min (entry 4, Table 3).

PD:

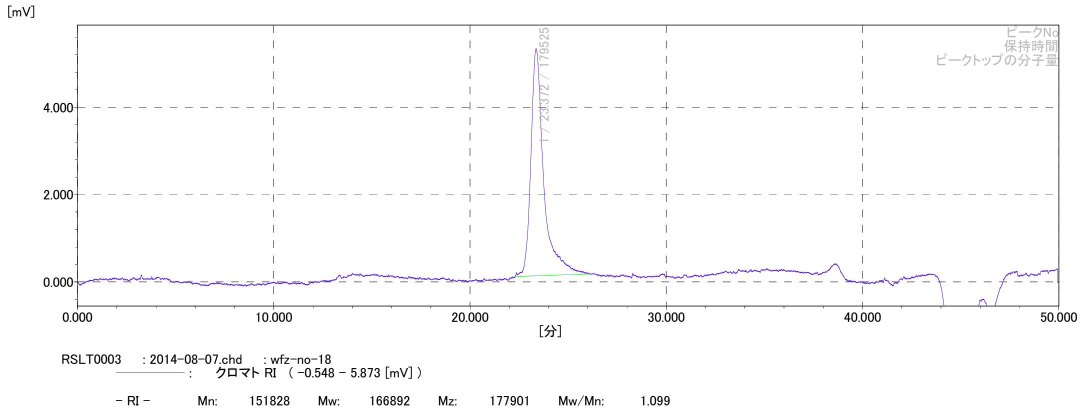

Figure S41. GPC trace for the poly(1-decene) obtained with **C1**-MMAO at 20 °C for 30 min (entry 5, Table 3).

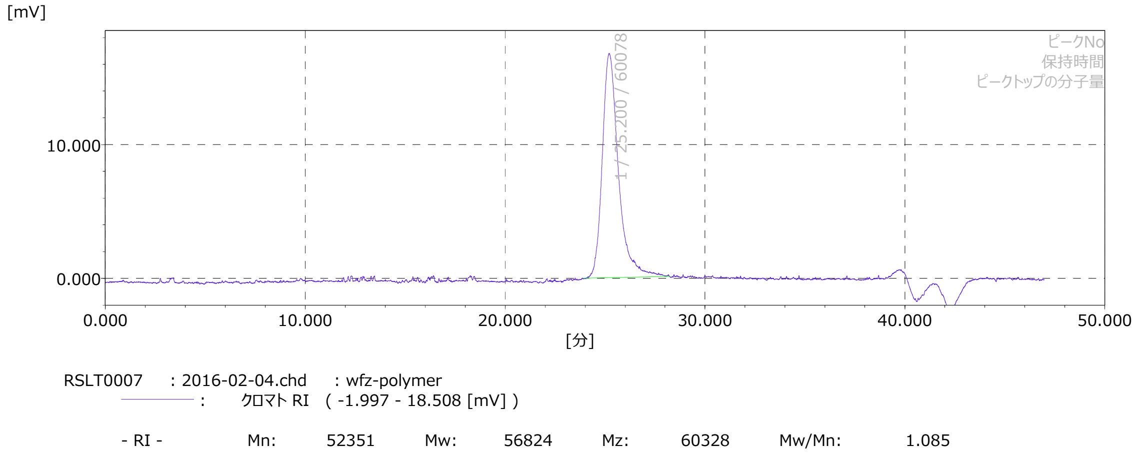

Figure S42. GPC trace for the poly(1-decene) obtained with **C1**-MMAO at 0 °C for 30 min (entry 7, Table 3).

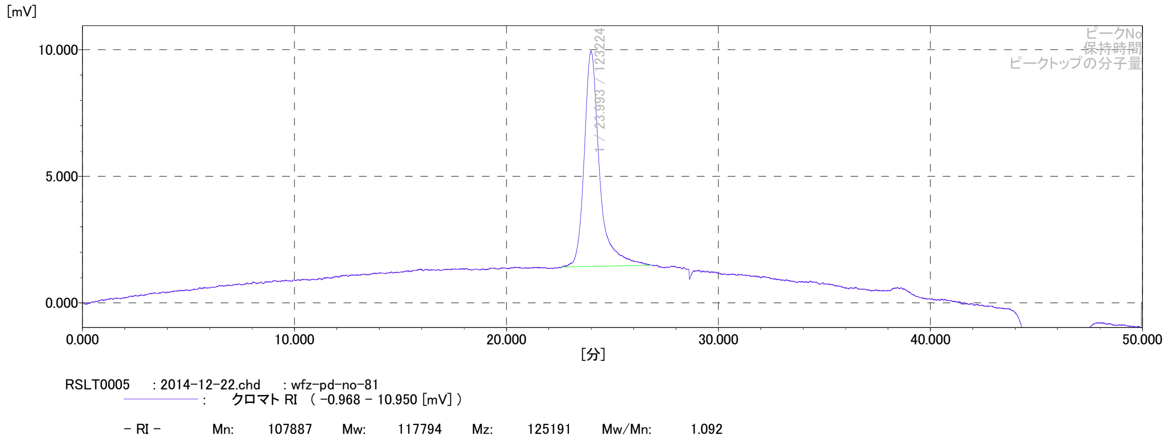

Figure S43. GPC trace for the poly(1-decene) obtained with **C1**-MMAO at 20 °C for 30 min (entry 8, Table 3).

PHD:

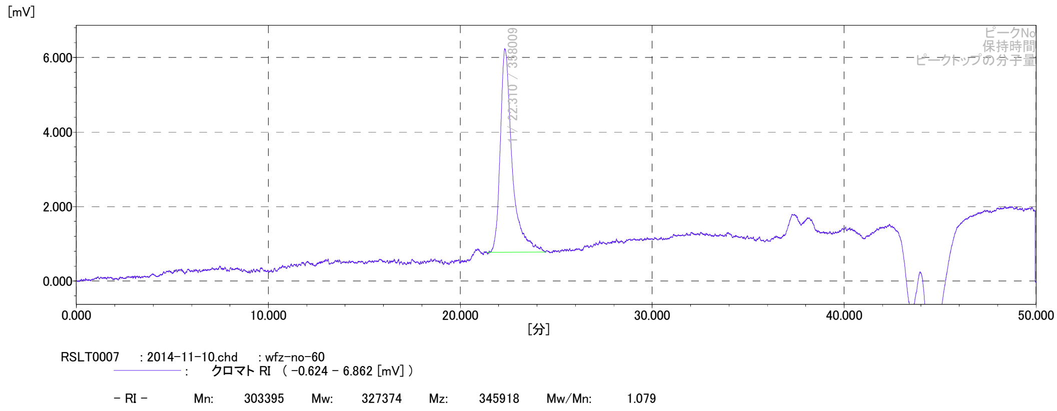

Figure S44. GPC trace for the poly(1-hexadecene) obtained with **C1**-MMAO at 20 °C for 30 min (entry 12, Table 3).

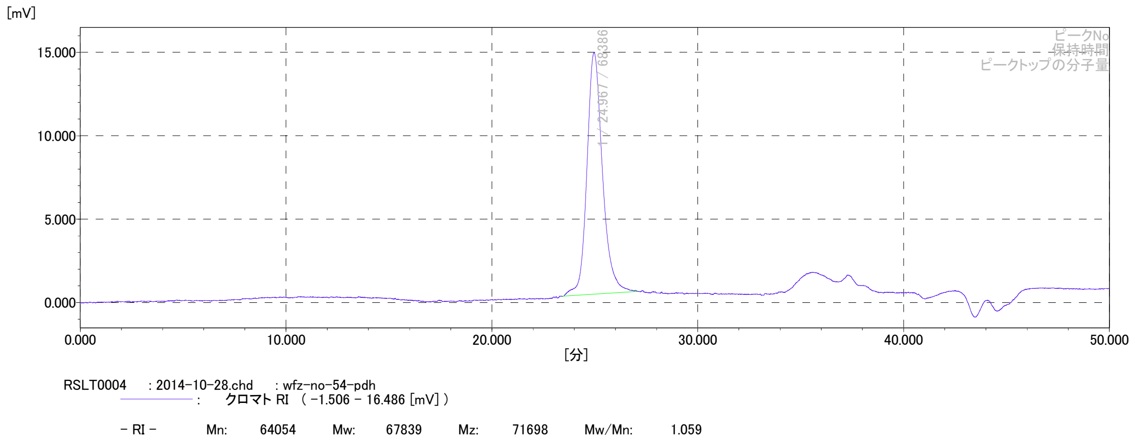

Figure S45. GPC trace for the poly(1-hexadecene) obtained with **C1**-MMAO at 0 °C for 30 min (entry 13, Table 3).

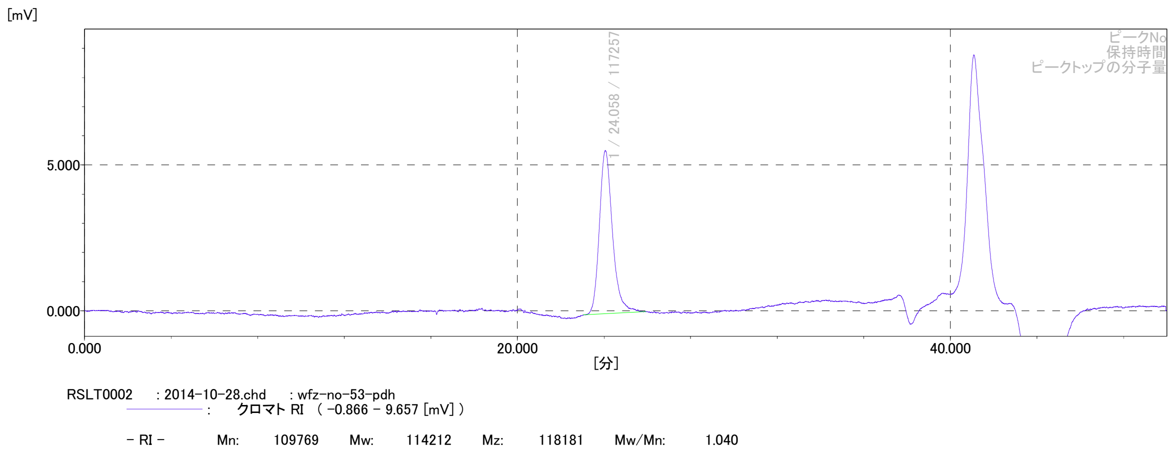

Figure S46. GPC trace for the poly(1-hexadecene) obtained with **C1**-MMAO at 20 °C for 30 min (entry 14, Table 3).

5. DSC Curves of the Polymers

PE:

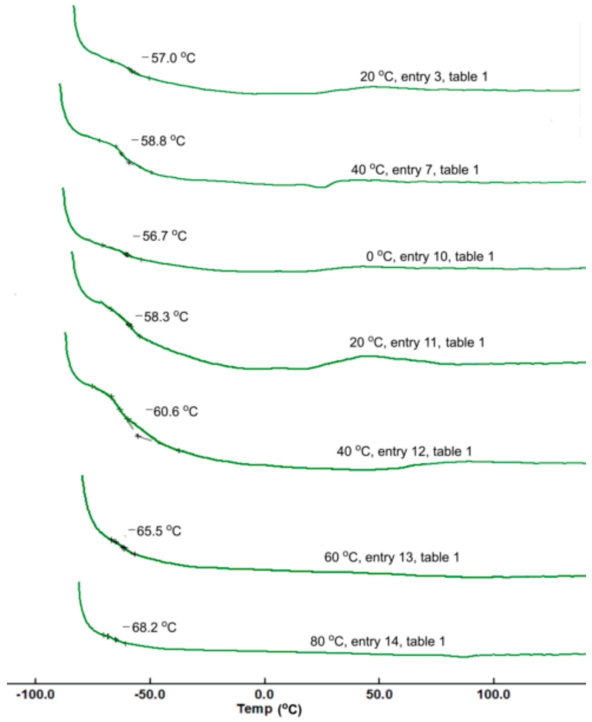

Figure S47. DSC curves of polyethylenes obtained by **C1**-MMAO at different temperatures (entries 3, 7 and 13–14, Table 1).

PH:

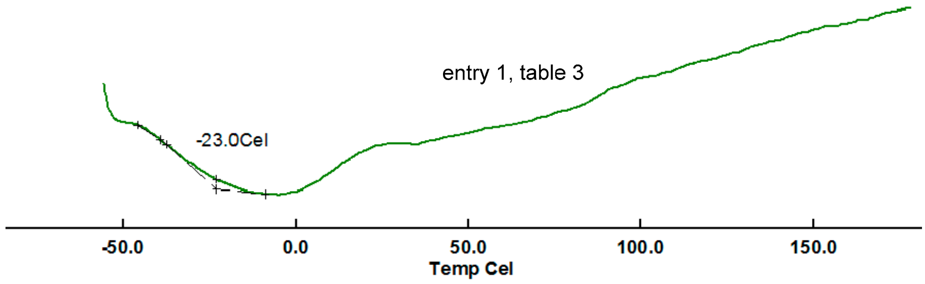

Figure S48. DSC curve for the poly poly(1-hexene) obtained with **C1**-MMAO at 20 °C (entry 1, Table 3).

PO:

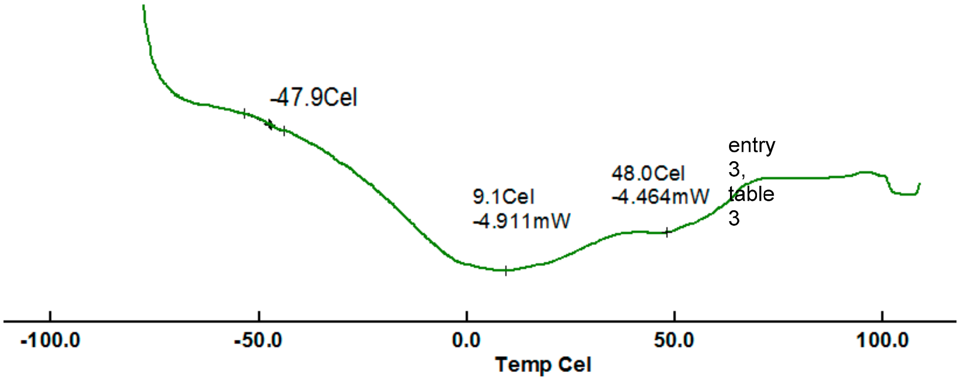

Figure S49. DSC curve for the poly(1-octene) obtained with **C1**-MMAO at 20 °C (entry 3, Table 3).

PD:

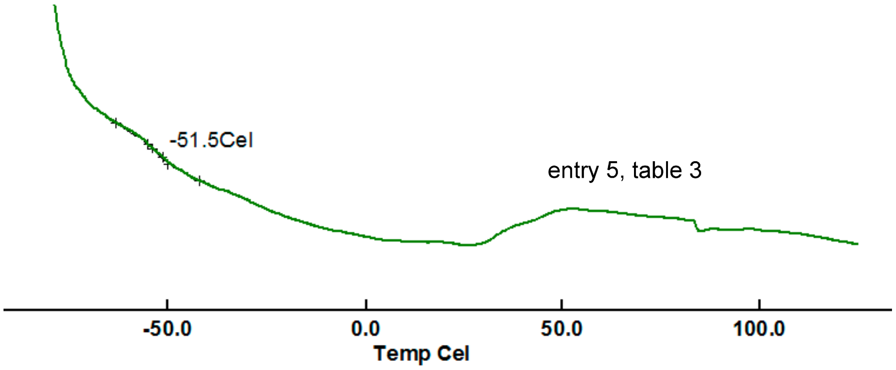

Figure S50. DSC curve for the poly(1-decene) obtained with **C1**-MMAO at 20 °C (entry 5, Table 3).

PHD:

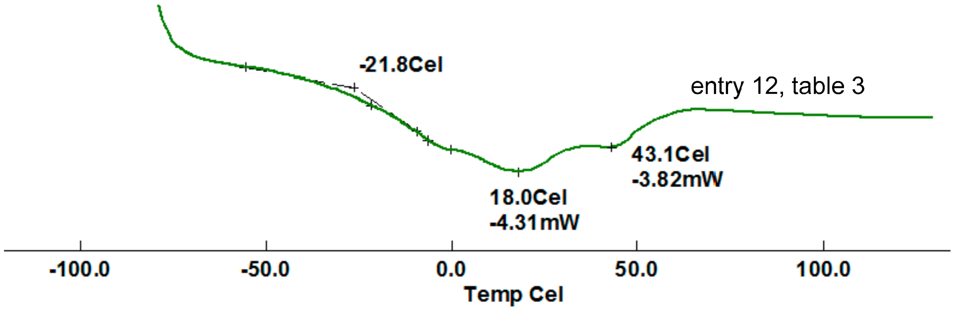

Figure S51. DSC curve for the poly(1-hexadecene) obtained with **C1**-MMAO at 20 °C (entry 12, Table 3).

References

1. McCord, E.F.; McLain, S.J.; Nelson, L.T.J.; Ittel, S.D.; Tempel, D.; Killian, C.M.; Johnson, L.K.; Brookhart, M. ^{13}C NMR Analysis of α-olefin enchainment in poly(α-olefins) produced with nickel and palladium α-diimine catalysts. *Macromolecules* **2007**, *40*, 410–420.
2. Azoulay, J.D.; Bazan, G.C.; Galland, G.B. Microstructural characterization of poly(1-hexene) obtained using a nickel α-Keto-β-diimine initiator. *Macromolecules* **2010**, *43*, 2794–2800.

© 2016 by the authors; licensee MDPI, Basel, Switzerland. This article is an open access article distributed under the terms and conditions of the Creative Commons Attribution (CC-BY) license (http://creativecommons.org/licenses/by/4.0/).
